# Identification of plant genes putatively involved in the perception of fungal ergosterol‐squalene

**DOI:** 10.1111/jipb.12862

**Published:** 2019-12-30

**Authors:** Laura Lindo, Rosa E. Cardoza, Alicia Lorenzana, Pedro A. Casquero, Santiago Gutiérrez

**Affiliations:** ^1^ Area of Microbiology University of León, Campus of Ponferrada Ponferrada Spain; ^2^ University Group of Research in Engineering and Sustainable Agriculture University of León León Spain

## Abstract

*Trichoderma* biocontrol strains establish a complex network of interactions with plants, in which diverse fungal molecules are involved in the recognition of these fungi as nonpathogenic organisms. These molecules act as microbial‐associated molecular patterns that trigger plant responses. Previous studies have reported the importance of ergosterol produced by *Trichoderma* spp. for the ability of these fungi to induce plant growth and defenses. In addition, squalene, a sterol biosynthetic intermediate, seems to play an important role in these interactions. Here, we analyzed the effect of different concentrations of ergosterol and squalene on tomato (*Solanum lycopersicum*) growth and on the transcription level of defense‐ and growth‐related genes. We used an RNA‐seq strategy to identify several tomato genes encoding predicted pattern recognition receptor proteins or WRKY transcription factors, both of which are putatively involved in the perception and response to ergosterol and squalene. Finally, an analysis of *Arabidopsis thaliana* mutants lacking the genes homologous to these tomato candidates led to the identification of a WRKY40 transcription factor that negatively regulates salicylic acid‐related genes and positively regulates ethylene‐ and jasmonate‐related genes in the presence of ergosterol and squalene.

## Graphical abstract

The relative concentration of ergosterol and squalene in the fungal mycelium is a critical parameter determining the plant responses. Analysis of plant genes involved in response to these metabolites led to the identification of a WRKY40 transcription factor that regulates plant hormone‐related genes in the presence of these compounds.

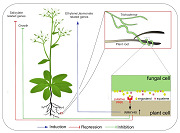

## INTRODUCTION

*Trichoderma* is a fungal genus distributed all over the world. It contains a remarkable number of strains able to act as biocontrol agents, which are characterized by their ability to antagonize plant‐pathogenic fungi and promote plant growth and defense responses (Druzhinina et al. 2011). *Trichoderma* biocontrol species have been extensively characterized, and this genus serves as a model for the study of beneficial fungus–plant interactions (Lorito et al. 2010).

Some *Trichoderma* strains protect plants against pathogenic fungi by producing antibiotics, hydrolytic enzymes, and/or other antifungal compounds (Lorito et al. 1996; Harman et al. 2004; Shoresh et al. 2010; Viterbo and Horwitz 2010; Hermosa et al. 2013). Indeed, *Trichoderma* establishes a signaling network with plants using diverse molecules that allow the perception of this fungus as a nonpathogenic organism. These molecules include microbial‐associated molecular patterns (MAMPs), pathogen‐associated molecular patterns (PAMPs), and damage‐associated molecular patterns (DAMPs). The latter are products of fungal hydrolytic enzymes, the function of which is to degrade plant cell walls (Hermosa et al. 2013). Microbial‐associated molecular patterns, PAMPs, and DAMPs are recognized by pattern recognition receptors (PRRs) in the plant. These receptors have an ectodomain, a single transmembrane domain, and a kinase endodomain, and activate a signaling cascade that triggers the plant defense response. This response, known as pattern‐triggered immunity (PTI), is a type of basal immunity providing resistance to a wide variety of pathogens (Macho and Zipfel 2015; Oren et al. 2016). In addition to PTI, plants also display effector‐triggered immunity, involving intracellular receptors able to directly or indirectly perceive secreted virulence effectors (Jones and Dangl 2006; Hermosa et al. 2013; Buchanan et al. 2015; Oren et al. 2016; Gust et al. 2017).

The ability of some *Trichoderma* strains to antagonize other pathogenic fungi is based on the production of a great variety of secondary metabolites, some of which exhibit antifungal activity (Sivasithamparam and Ghisalberti 1998; Hermosa et al. 2013). Among them, the trichothecenes have attracted much attention due to their roles as mycotoxins affecting plants and animals, including humans (McCormick et al. 2011). Furthermore, the trichothecene biosynthetic pathway is important in maintaining the balance of terpene intermediates, as well as the levels of ergosterol (Malmierca et al. 2015a, 2015b), which is the main sterol in fungi and is perceived as a nonself molecule by plants, triggering a plant defense response (Rossard et al. 2010; Malmierca et al. 2015c). Indeed, there is increasing evidence to suggest the importance of ergosterol in the ability of *Trichoderma* to induce plant growth and defense (Malmierca et al. 2015c). Ergosterol is also considered a PAMP / MAMP (Vatsa et al. 2011) and causes: (i) changes in the membrane potential (Rossard et al. 2006, 2010); (ii) the modification of H^+^ fluxes; and (iii) the production of active oxygen species (Kasparovsky et al. 2003). Based on these findings, it is hypothesized that ergosterol must be perceived by PRRs located in the plant cell membrane, although these have not yet been identified.

Some PRRs are characterized by leucine‐rich repeat (LRR) motifs (Jones and Dangl 2006; Gust et al. 2017), which comprise 20 to 30 amino acids with a conserved sequence: LccLcLccNxL/LxxLxLxxCxxL (L = Val, Leu, or Ile; N = Asn, Thr, Ser, or Cys; C = Cys or Ser). Pattern recognition receptors perceive PAMPs or MAMPs and trigger several molecular pathways (Enkhbayar et al. 2004), in which transcription factors (TFs) play an important role. The WRKY domain‐containing TFs, which have the conserved motif WRKYGQK at their N‐terminal end and a zinc‐finger‐like motif at their C‐terminal end (Bai et al. 2018), have been reported to be involved in the signal transduction cascades in plant‐microbe interactions (Eulgem et al. 2000) and could therefore be involved in the response to ergosterol.

In addition to ergosterol, some intermediates of its biosynthesis, for example squalene, play a role in fungus‐plant interactions. This intermediate is usually located in the plasma membrane, but also accumulates in the cytoplasm as lipid droplets (Zweytick et al. 2000). Previous reports indicate that the relative levels of these two compounds, that is ergosterol to squalene, seem to be important for some plant responses against fungi, and they strongly determine the fluidity or rigidity of the fungal cell membranes (Spanova et al. 2012), thereby determining the efficiency of fungal metabolite exchange with the environment. The relative ergosterol to squalene ratio has also been reported to affect the ability of *Trichoderma* to colonize tomato (*Solanum lycopersicum*) plant roots and to influence the expression of genes involved in the salicylic acid (SA) and jasmonate/ethylene (JA/ET) pathways, which are both related to plant defense responses (Garaiová et al. 2014; Cardoza et al. 2015).

In this work, we used *in vitro* and *in vivo* approaches to analyze the effect of fungal ergosterol and squalene levels in plant growth and defense responses. Furthermore, using an RNA‐seq strategy, we identified tomato genes likely to be involved in the perception of ergosterol/squalene, and determined how *Arabidopsis thaliana* knockout mutants of the homologs of some of these tomato genes respond to the presence of ergosterol and squalene in the growth media. As a result, we have identified an *A. thaliana* WRKY40 transcription factor involved in this response that negatively regulates SA‐related genes and positively regulates ET‐ and JA‐related genes in the presence of ergosterol and squalene.

## RESULTS

### High ergosterol combined with low squalene concentrations have the largest phenotypic effects on tomato plants

To determine the ratios of ergosterol/squalene with the highest effect on tomato growth, tomato plants were grown in four different concentrations of ergosterol and squalene corresponding to those produced by *Trichoderma harzianum* CECT 2413 (T34‐wild‐type) and mutants derived from this strain: T34‐E20, T34‐E1.33, and T34‐SIL.E7 (Table 1) and were compared to plants grown in the control condition without ergosterol or squalene (Figure S1). The ergosterol and squalene concentrations corresponding to those in T34 positively affected root growth, despite there being no apparent differences in the aboveground parts. The roots grown in the T34 ergosterol/squalene condition were longer and formed more secondary roots (Figure S1B). There are no visual differences between the plants grown in the control condition *versus* those grown in the condition with the T34‐SIL.E7 ergosterol/squalene concentrations (Figure S1E). In the other two conditions, T34‐E20 and T34‐E1.33 (Figure S1C, D), the aerial shoots and the roots were smaller than those grown in the control condition, and fewer secondary roots were produced. Based on these data, we selected the ergosterol/squalene concentrations simulating those of T34 (0.033 mg/mL ergosterol and 0.6 µg/mL squalene) for experimental use.

**Table 1:** Volumetric production of ergosterol and squalene by the strains *Trichoderma harzianum* CECT 2413 (T34‐wild‐type) and mutants derived from this strain: T34‐E20, T34‐E1.33, and T34‐SIL.E7

Strain	Squalene (µg/mL)	Ergosterol (mg/mL)
T34	0.60	0.033
T34‐E20	3.00	0.022
T34‐E1.33	0.13	0.034
T34‐SIL.E7	0.21	0.014

### Ergosterol/squalene at the concentrations produced by the T34 strain upregulate tomato genes related to the JA/ET and nitrogen assimilation pathways

**Figure 1:**
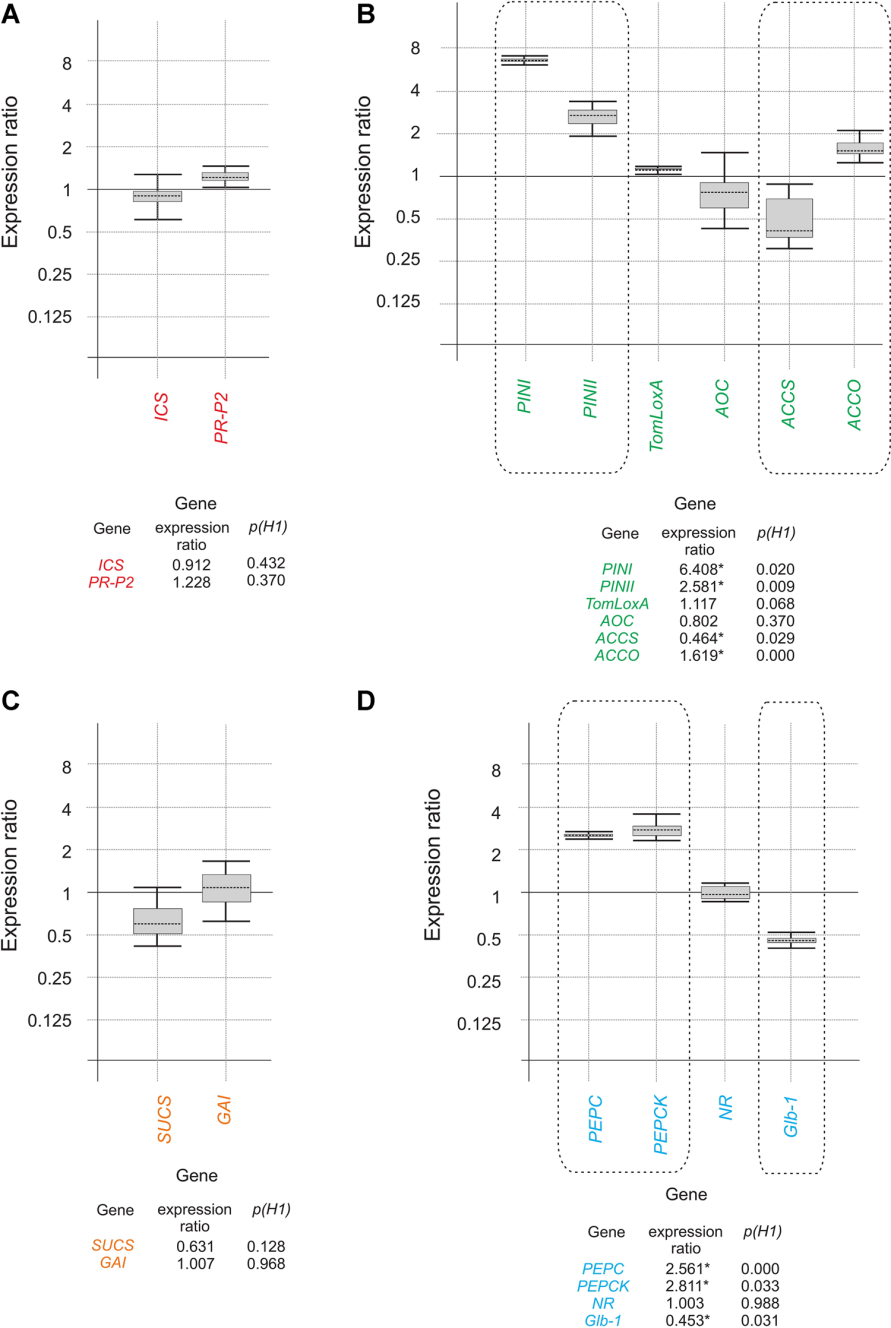
**Effect of tomato plants growth on ergosterol/squalene at T34 concentrations in the expression of tomato defense‐ and growth‐related genes** Real‐time quantitative polymerase chain reaction (RT‐qPCR) analysis of the expression ratios of four groups of genes between tomato plants grown in the presence of ergosterol/squalene concentrations simulating those produced by the T34 strain of *Trichoderma versus* the expression in the control condition lacking these exogenous compounds. **(A)** Genes related to the salicylic acid (SA) signaling and biosynthetic pathways (red). **(B)** Genes related to the jasmonate/ethylene (JA/ET) signaling and biosynthetic pathways (green). **(C)** Growth‐related genes (orange). **(D)** Genes involved in nitrogen assimilation (blue). The RT‐qPCR data were analyzed using REST software (Pfaffl et al. 2002). Statistically significant values (p(H1) < 0.05) are indicated with an asterisk in the tables below the graphs and surrounded with a dotted rectangle in the graphical representation. Three biological replicates with four plants per replicate were used.

We performed a real‐time quantitative polymerase chain reaction (RT‐qPCR) analysis to compare the transcription levels of genes related to plant defense, hormone biosynthesis, growth, and nitrogen assimilation in the roots of tomato plants grown in the presence of the T34 ergosterol/squalene concentrations *versus* control plants grown in media amended only with the solvents but without these compounds. Among the 14 genes analyzed (see Materials and Methods section for details), five were upregulated; these belonged to the JA/ET signaling and biosynthetic pathways (*PINI*, *PINII*, and *ACCO*) as well as being involved in nitrogen assimilation (*PEPC* and *PEPCK*). Furthermore, only two genes were significantly downregulated, *ACCS* and *Glb‐1*, which encode enzymes involved in hormone biosynthesis and nitrogen assimilation, respectively (Figure 1). This observed upregulation of genes related to the JA/ET pathways and nitrogen assimilation, along with the lack of effect on genes related to SA signaling and growth pathways, suggests a role for ergosterol/squalene as MAMPs that would trigger these plant responses.

### Tomato plants grown in the presence of T34 and T34‐E20 mycelia exhibited similar responses to those grown with pure ergosterol/squalene at the T34 and T34‐E20 concentrations

**Figure 2:**
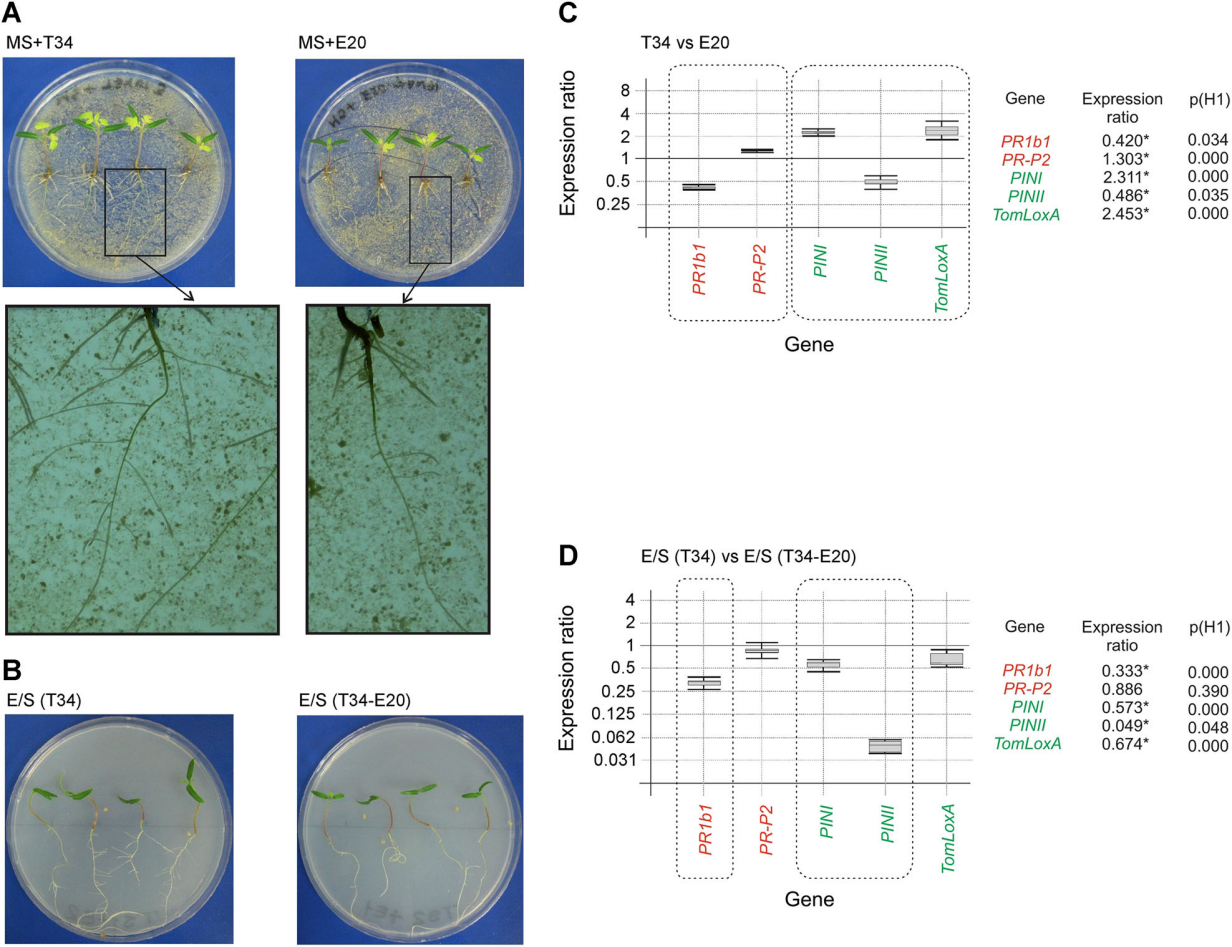
**Effect of T34 and T34‐E20 mycelia and the ergosterol/squalene concentrations produced by these fungal strains in tomato plants growth and in the expression ratio of defense‐related genes** Effect of **(A)** the mycelia of T34 and T34‐E20 or **(B)** the different concentrations of squalene (S)/ergosterol (E) used in the growth of tomato plants. **(C, D)** Real‐time quantitative polymerase chain reaction (RT‐qPCR) analysis of the expression of defense‐related genes, related to salicylic acid (SA)‐ (*PR1b1* and *PR‐P2*) (red) or to the jasmonate/ethylene (JA/ET)‐ (*PINI, PINII* and *TomLoxA*) (green) signaling pathways. Note that E/S (T34) and E/S (T34‐E20) reflect the concentrations of E and S detected in the T34 and T34‐E20 mycelia, respectively. Three biological replicates were used for each condition. The expression studies and graphical representation were performed as described in the legend to Figure 1.

Ergosterol and squalene are usually localized in the fungal membranes, from where they might interact with plant proteins, that is receptors, that would perceive these compounds and activate the plant response. Thus, we hypothesized that the use of fungal mycelia to supplement the growth medium could itself activate the plant response, on the basis of the localization of ergosterol/squalene, as structural components, in the fungal cell outer layers. Following this rationale, and in order to compare the effects of fungal mycelia *versus* those observed with pure ergosterol and squalene, an experiment was performed in which tomato plants were grown on 150‐mm plates containing 100 mL of solid Murashige‐Skoog (MS) medium amended with 0.4 g of freeze‐dried T34 or T34‐E20 mycelia. These two strains were selected because they showed a clearly different pattern of ergosterol/squalene production (Table 1). The concentration of ergosterol/squalene in the media amended with fungal mycelia had a ratio similar to those previously quantified in the fungal mycelia (Table S1). After 21 d of growth in these media, the phenotypes of the plants were analyzed along with their level of tomato defense‐related gene expression. Remarkable differences were observed between the tomato phenotypes; plants grown in the presence of T34 mycelia exhibited more growth in both the shoot and root, including a higher number of secondary roots, than the plants grown in the medium supplemented with T34‐E20 mycelia (Figure 2A). These results were consistent with those observed when the plants were grown in exogenous ergosterol/squalene (Figures 2B, S1).

**Figure 3:**
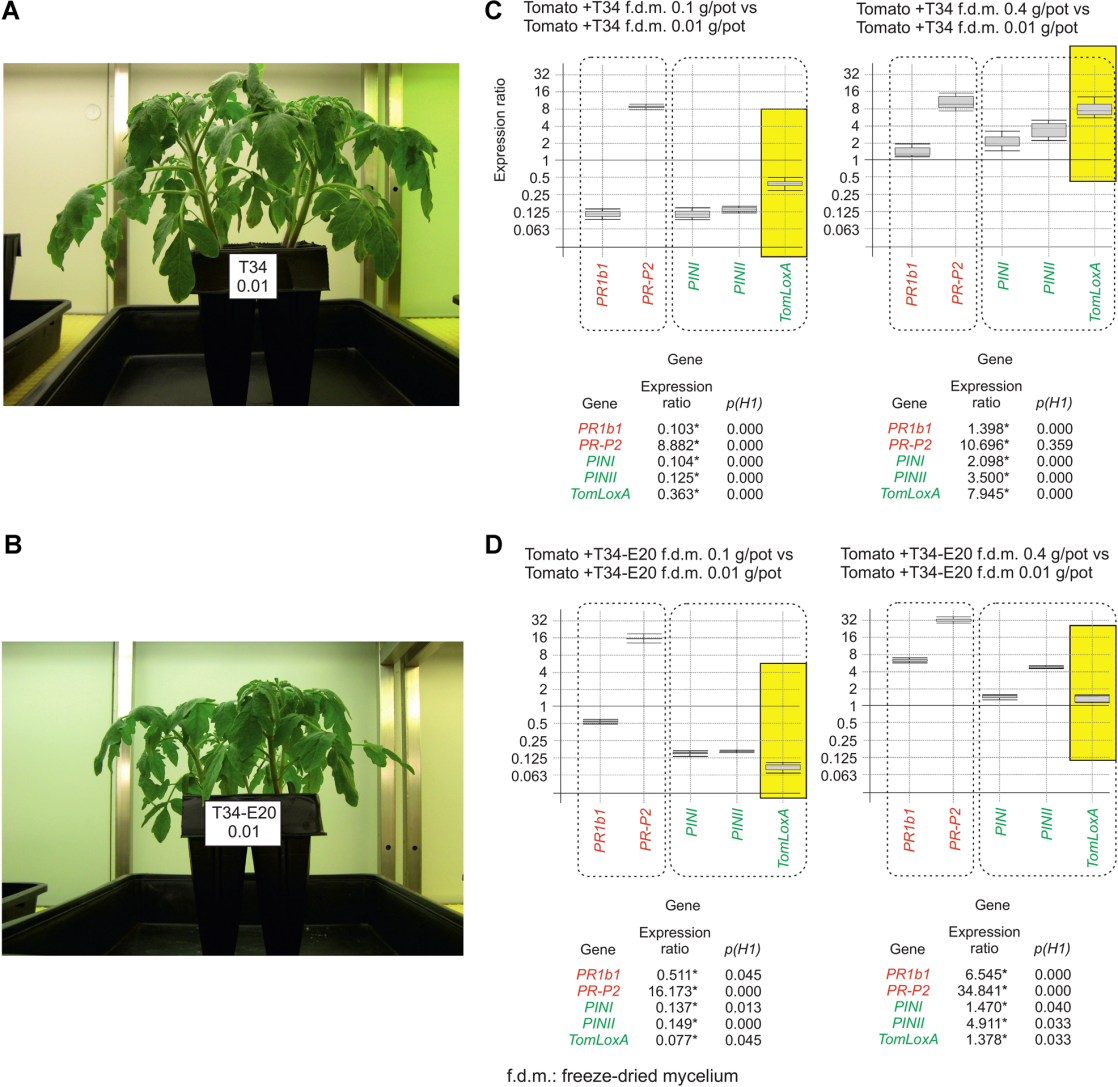
***In vivo* effect of *Trichoderma* mycelia on tomato plant growth and in the regulation of plant defense‐related genes** **(A, B)** Tomato plants grown for 4 weeks on substrate amended with freeze‐dried mycelia (0.01 g/pot) from T34 **(A)** or T34‐E20 **(B)**. **(C, D)** Real‐time quantitative polymerase chain reaction (RT‐qPCR) analysis of the expression of tomato defense‐related genes (salicylic acid (SA)‐related genes (red); jasmonate/ethylene (JA/ET)‐related genes (green)); in plants grown in media amended with different amounts of freeze‐dried mycelia from T34 **(C)** or T34‐E20 **(D)**. Yellow rectangles were used to highlight the levels of expression observed for *TomLoxA*. Eight plants were analyzed for each condition. A brief description of the genes analyzed by RT‐qPCR was included in the legend to Figure 2. Graphical representation of the expression ratio values as described in the legend to Figure 1.

An analysis of the expression ratios of genes related to the SA signaling (*PR1b1* and *PR‐P2*) and JA/ET signaling pathways (*PINI*, *PINII*, and *TomLoxA*), in the aerial parts of plants grown in the presence of the T34 or T34‐E20 mycelia, led to the observation that there is not a clear pattern for all genes belonging to any particular group. *PR1b1* and *PR‐P2*, both belonging to the SA signaling pathway, were down‐ and upregulated, respectively, and a similar result was observed for the JA/ET‐related genes. The most remarkable result was that the pattern of expression ratios for all these genes in the presence of T34 or T34‐E20 mycelia was very similar to that observed when the plants were grown in the presence of pure ergosterol and squalene at concentrations representative of these strains (Figure 2C, D). These results led us to conclude that both the phenotypic and transcriptomic effects observed when using mycelia from the T34 or T34‐E20 strains were due to their ergosterol and squalene contents, which likely interact with the plant receptors to initiate a response, in this case stimulating growth (T34 mycelium) and affecting the expression of plant defense‐related genes. Furthermore, an *in vivo* assay was performed to determine the effect of these mycelia on tomato plant growth and compare the results with those of the *in vitro* assays. In the presence of the T34 mycelia, the tomato plants grew much faster, particularly in their root systems (Figures 3A, S2), which correlates with the results observed in the *in vitro* assays.

Real‐time quantitative polymerase chain reaction analysis revealed that SA‐related genes were generally upregulated in plants grown in the presence of T34‐E20 mycelia compared to the relative levels obtained when using T34 mycelia. No remarkable differences were observed in the same comparison between the JA/ET‐related genes, except for *TomLoxA* (Figure 3B, yellow box), which exhibited higher levels of expression when the T34 mycelia were used. *TomLoxA* is believed to be associated with the control of the spread of beneficial fungi through the roots (León‐Morcillo et al. 2012). The increased expression of this gene in plants grown in the presence of T34 mycelia indicates that, in addition to the observed phenotypic changes, higher concentrations of ergosterol and lower concentrations of squalene in the *Trichoderma* membranes (such as those observed in the T34 mycelia) promote the fungal colonization of the plant roots.

### Identification of three genes, encoding two PRR and a WRKY transcription factor, that were strongly upregulated in tomato plants grown in the presence of ergosterol/squalene at T34 concentrations

We conducted an RNA‐seq analysis to determine the effect of growth in the presence of the selected T34 concentrations of ergosterol/squalene on the tomato transcriptome. A total of 1,147 genes (3.2% of the 35,567 tomato transcripts) were differentially expressed in tomato plants grown with exogenous ergosterol/squalene compared to the level of expression in plants grown in the control conditions. Among the differentially expressed genes (DEGs), we searched for genes encoding proteins containing LRR or WRKY domains, which might directly bind to ergosterol and/or squalene (potential PRR receptors containing LRR domains) or regulate the plant response to these compounds by acting as TFs (WRKY domain‐containing proteins). This resulted in the identification of 65 ergosterol‐ and/or squalene‐related genes putatively encoding 58 LRR proteins and seven WRKY proteins.

To refine the search, we selected only those genes showing a two‐fold upregulation or greater. A total of 23 of the 58 upregulated genes encoding for LRR domain‐containing proteins matched this criterion (Table 2), and two with the higher ratio values, XP_004250531.1 and XP_004238856.2, were selected for further experiments exploring their putative roles as PRRs. For the genes encoding WRKY domain‐containing proteins, XP_004245210.1 and NP_001304843.1 were selected from the five genes with a two‐fold upregulation or greater (Table 3).

**Table 2:** Basic Local Alignment Search Tool analysis of the leucine‐rich repeat (LRR) domain‐containing proteins encoded by genes upregulated in tomato plants treated with ergosterol and squalene (TRS1E2) *versus* untreated plants (TR)

Protein	Ratio TRS1E2/TR	Description	E value	Accession
Solyc01g067020	3.08	Atypical receptor‐like kinase 1 precursor	0.0	NP_001234580.2
Solyc01g102675	2.38	Probable LRR receptor	6E‐84	XP_010314121.1
Solyc01g102680	2.31	Probable LRR receptor	0.0	XP_010314121.1
Solyc02g072470	2.79	Probable LRR receptor	0.0	XP_010316829.1
Solyc04g009640	2.58	Receptor kinase‐like protein Xa21	0.0	XP_004237164.2
Solyc04g054445	2.52	Receptor‐like protein 30‐like	0.0	XP_004237609.2
Solyc06g033920	2.02	Receptor‐like protein 30‐like	0.0	XP_004240813.1
Solyc07g005150	2.36	Receptor‐like protein 12	0.0	XP_015081177.1
Solyc07g006480	2.95	Probably inactive leucine	0.0	XP_015081747.1
Solyc07g006770	2.37	Probable LRR receptor	0.0	XP_004242680.2
Solyc07g008590	2.28	LRR receptor	0.0	XP_010323339.1
Solyc09g082530	2.86	LRR extensin‐like protein 6	0.0	XP_004247590.1
**Solyc11g011180**	**6.37**	**Probable LRR receptor**	**0.0**	**XP_004250531.1**
Solyc11g007790	3.04	Probable disease resistance protein	0.0	XP_004250491.1
Solyc03g006730	2.11	G‐type lectin S‐receptor	0.0	XP_015162372.1
Solyc03g006890	3.97	PTI1‐like tyrosine‐protein kinase	0.0	XP_004234145.1
Solyc03g059080	2.16	Probable protein kinase	0.0	XP_004234864.1
**Solyc05g008310**	**4.05**	**G‐type lectin S‐receptor**	**0.0**	**XP_004238856.2**
Solyc07g053130	3.00	G‐type lectin S‐receptor	0.0	XP_010323780.1
Solyc09g014720	2.80	Wall‐associated receptor kinase 2‐like	0.0	XP_004246830.2
Solyc09g090210	3.32	Probable protein kinase	0.0	XP_004247739.1
Solyc10g006720	2.09	G‐type lectin S‐receptor	0.0	XP_019071434.1
Solyc09g014730	2.04	Wall‐associated receptor kinase 2‐like	0.0	XP_010325785.1

Note: only genes with expression ratios greater than 2 are included. The selected genes, exhibiting the highest ratios of expression, are highlighted in bold type. TR, roots from untreated plants; TRS1E2, roots from squalene and ergosterol concentrations produced by T34 (S1 = low squalene concentration, and E2 = high ergosterol concentration).

**Table 3:** Basic Local Alignment Search Tool analysis of WRKY domain‐containing proteins encoded by genes upregulated in tomato plants treated with ergosterol and squalene (TRS1E2) *versus* untreated plants (TR)

Protein	Ratio TRSIE2/TREA	Description	E value	Accession
Solyc05g015850	2.18	Probable WRKY transcription factor 75	2E‐126	XP_015075730.1
**Solyc08g067340**	**5.53**	**Probable WRKY transcription factor 40**	**0.0**	**XP_004245210.1**
**Solyc08g067360**	**8.63**	**Probable WRKY transcription factor 40**	**0.0**	**NP_001304843.1**
Solyc08g081610	2.17	Probable WRKY transcription factor 29	0.0	XP_004245564.1
Solyc04g051690	2.17	Probable WRKY transcription factor 51	8E‐92	XP_015160883.1

Note: only genes with expression ratios greater than 2 are included. The selected genes, exhibiting the highest ratios of expression, are highlighted in bold type. For details regarding the meaning of some abbreviations see Table 2.

### Mutants of *A. thaliana* in genes involved in the response to ergosterol/squalene show remarkable differences in the transcription level of defense‐ and development‐related genes in the presence of these compounds *versus* the wild‐type

To assess the involvement of the previously selected genes that were upregulated in tomato plants when grown in the presence of ergosterol/squalene at the T34 concentrations, three *A. thaliana* mutants, deleted in genes homologous to these tomato genes, were purchased from the European Arabidopsis Stock Center. *Arabidopsis thaliana* col‐0 was used as the wild‐type line. SALK_064666C (=α666C) contained a mutation in a gene homologous to one encoding XP_004250531.1 in tomato, SALK_129987C (=α987C) contained a mutation in a gene encoding a XP_004238856.2 homolog, and SALK_0398333.1 (=α331) contained a mutation in a gene encoding a NP_001304843.1 homolog. No homolog was identified in the *A. thaliana* genome for the tomato gene encoding XP_004245210.1; thus, this gene was discarded from further experiments.

**Figure 4:**
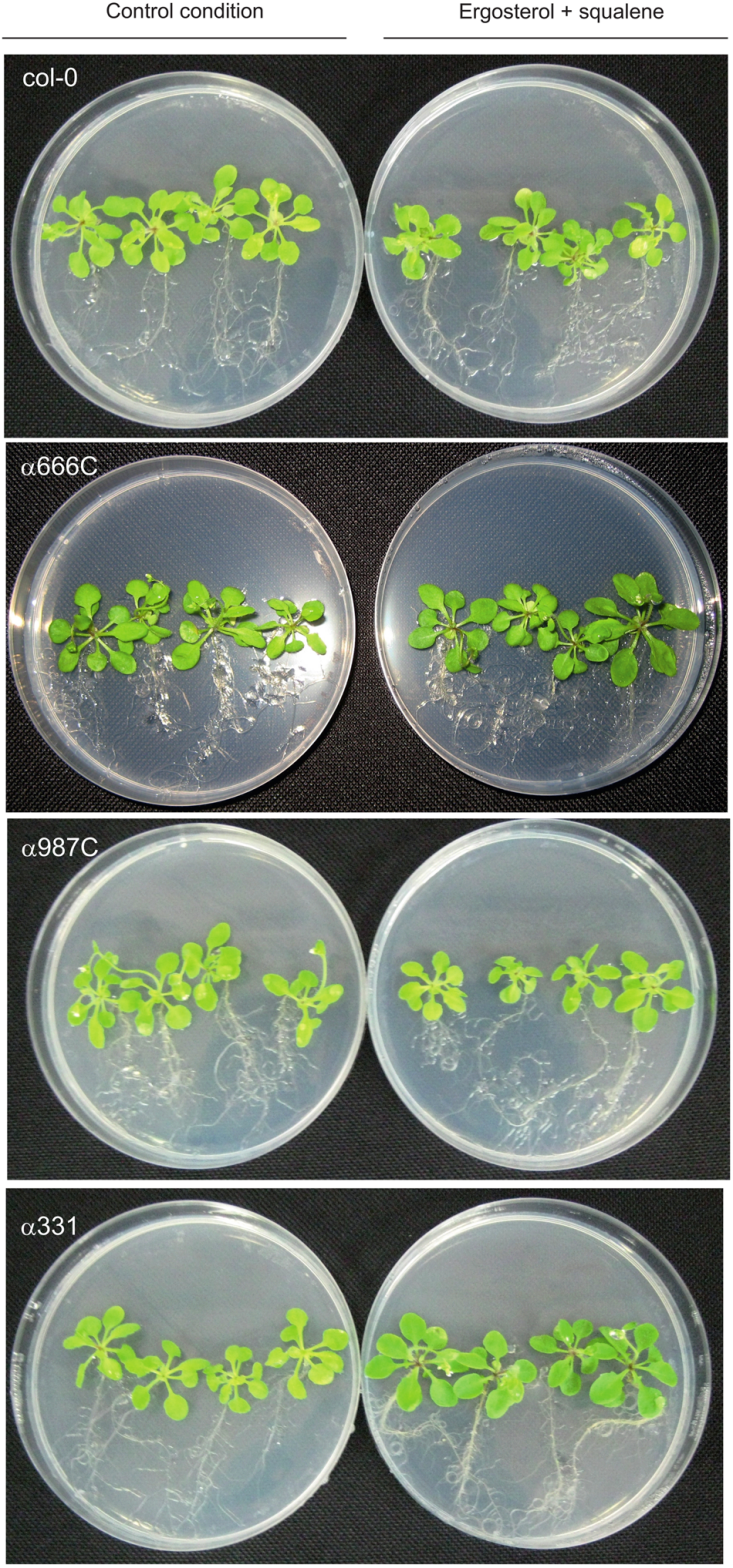
**Effect of ergosterol/squalene on growth of *Arabidopsis thaliana* varieties that were mutated on genes putatively involved in the plant perception and response to these compounds** Lines analyzed: *A. thaliana* col‐0 (wild‐type), and mutants α666C, α987C, and α331 grown for 7 d with (right panels) or without (left panels; control condition) the T34 concentrations of ergosterol/squalene. Three different biological replicates were used for each combination of plant line and condition.

The mutants and col‐0 were grown in media with or without the T34 concentrations of ergosterol/squalene. A slight increase in the growth of the aerial tissues was observed in the col‐0 plants in the absence of ergosterol and squalene compared with plants grown with these compounds (Figure 4), while a slight increase and reduction in the growth of the aerial parts were observed in the α666C and α987C mutants, respectively, in the presence of ergosterol/squalene (Figure 4). The most remarkable data were the increased development of the aerial parts and in the thickness and number of secondary roots in α331 plants growing with ergosterol/squalene, compared to plants in the control condition (Figure 4).

**Figure 5:**
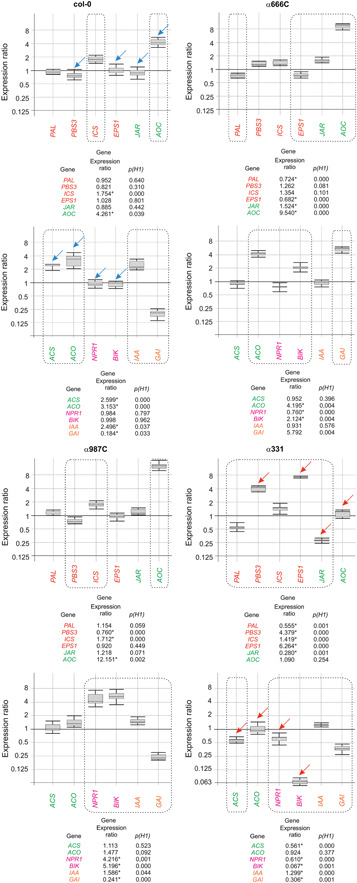
**Effect of ergosterol/squalene on the transcription level of *Arabidopsis thaliana* genes related to defense and growth in the four varieties selected in the present work** Real‐time quantitative polymerase chain reaction (RT‐qPCR) analysis of the expression ratio of genes in the *A. thaliana* lines col‐0, α666C, α987C, and α331 grown in the presence of exogenous ergosterol/squalene, relative to their expression in plants grown in the control condition, without ergosterol/squalene. Four groups of genes were analyzed: (i) salicylic acid (SA)‐related genes (red); (ii) jasmonate/ethylene (JA/ET)‐related genes (green); (iii) pathogenesis‐related genes (pink); and development‐related genes (orange). The calculation of the expression ratios and the graphic representations were performed as indicated in the legend to Figure 1. Arrows highlight the genes exhibiting the most remarkable differences in their expression ratios between the α331 mutant (red arrows) and col‐0 (blue arrows).

The transcription levels of various plant defense‐ and growth‐related genes (listed in Materials and Methods) were affected by the presence of ergosterol/squalene (Figure 5), but to varying degrees in the different plant lines analyzed, that is the wild‐type and mutants. The four SA biosynthesis genes analyzed (*PAL*, *PBS3*, *ICS*, and *EPS1*) only varied slightly in their expression ratios in α666C and α987C plants grown in the presence of ergosterol/squalene compared to their expression in the control plants (Figure 5). However, in the α331 plants *PBS3* and *EPS1* were strongly upregulated in the ergosterol/squalene condition, with expression ratios of 4.379‐fold (*p(H1)* = 0.000] and 6.264‐fold [*p(H1)* = 0.000), respectively, *versus* values of 0.821‐fold (*p(H1)* = 0.310) and 1.028‐fold (*p(H1)* = 0.801), respectively, in the col‐0 ecotype.

Regarding the genes belonging to the JA/ET pathways (*JAR*, *AOC*, *ACCS*, and *ACCO*), the α331 mutant again differed the most compared to the wild‐type line. All four genes were downregulated in α331 by the presence of ergosterol/squalene, with expression ratios significantly lower than those exhibited by col‐0 (Figure 5). Among the other two mutants analyzed, the most remarkable difference observed was the strong upregulation of *AOC* (JA/ET pathway) in the α987C and α666C mutants. These mutants exhibited a 12.151‐fold (*p(H1)* = 0.002) and 9.540‐fold (*p(H1)* = 0.000) increase in the expression of this gene under the ergosterol/squalene condition, respectively, *versus* a 4.261‐fold (*p(H1)* = 0.039) change in the wild‐type (Figure 5).

The behavior of the *PR* genes analyzed (*NPR1* and *BIK1*) was again remarkably different in α331 compared to the other strains. Both genes were downregulated in that mutant compared to col‐0, reaching expression ratios of 0.610‐fold (*p(H1)* = 0.000) and 0.067‐fold (*p(H1)* = 0.001), respectively, in α331 *versus* values of 0.984‐fold (*p(H1)* = 0.797) and 0.998‐fold (*p(H1)* = 0.962), respectively, in col‐0. Also, a remarkable upregulation of these two genes was observed in the α987C mutant, reaching values of 4.216‐fold (*p(H1)* = 0.001) and 5.196‐fold (*p(H1)* = 0.000), respectively (Figure 5).

Finally, two plant development genes (*IAA* and *GAI*) were analyzed. No significant differences were detected in the expression ratios of *IAA* between the strains when grown in the ergosterol/squalene or control conditions; however, *GAI* was strongly upregulated in strain α666C by 5.792‐fold (*p(H1)* = 0.004), compared to the 0.184‐fold (*p(H1)* = 0.033) change observed in col‐0. However, the differences in the expression ratios of this gene were lower than two‐fold in the other two mutants (α987C and α331) (Figure 5).

Summarizing these transcriptomic data, the α331 mutant exhibited the most remarkable differences among the three mutants analyzed, which were deleted in homologs to tomato genes previously selected on the basis of their upregulation in plants grown with ergosterol/squalene at the concentrations produced by the T34 strain. Therefore, this mutant was selected for further study.

### The deletion of the NP_001304843.1 homologous gene in the *A. thaliana* α331 mutant drastically affects the response of plants to the pathogen *Botrytis cinerea* B05.10 in the presence of ergosterol/squalene

To determine how the mutation in the NP_001304843.1 homologous gene, which alters the response of *A. thaliana* to ergosterol/squalene, affected the plant response to the pathogen *B. cinerea*, α331 and col‐0 plants grown with and without ergosterol/squalene were infected with *B. cinerea* B05.10 and grown for 96 h before being analyzed (Figure S3). The infection with B05.10 of plants grown in media with ergosterol/squalene negatively affected both strains of plants, with reduced growth of the roots and aerial parts. The *B. cinerea* damage was similar in plants grown in the absence of ergosterol/squalene.

**Figure 6:**
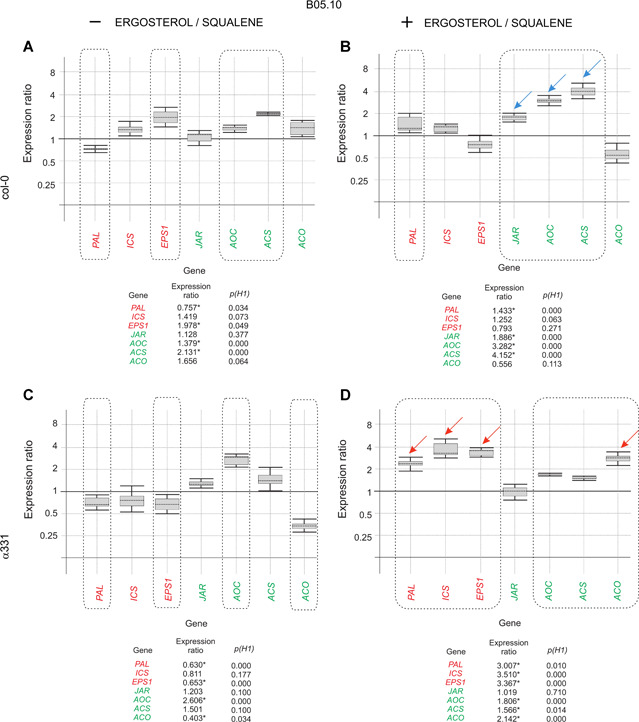
**Assessment of the response of the α331 mutated line against *Botrytis cinerea* in the presence of ergosterol/squalene, compared to the *Arabidopsis thaliana* col‐0 (wild‐type)** Real‐time quantitative polymerase chain reaction (RT‐qPCR) analysis of the expression of seven *A. thaliana* selected genes in plants infected with *B. cinerea* B05.10 and grown with or without ergosterol and squalene. **(A, C)** Expression ratios of the analyzed genes in plants infected with B05.10 and grown in Murashige–Skoog medium without ergosterol/squalene *versus* the levels of expression in plants noninfected with B05.10 and grown in the same conditions. **(B, D)** Expression ratios of the analyzed genes in plants infected with B05.10 and grown in media amended with ergosterol/squalene *versus* plants grown in the same conditions but noninfected with B05.10. **(A, B)** *A. thaliana* col‐0 plants. **(C, D)** α331 plants. The calculation of the expression ratios and graphic representations were performed as indicated in the legend to Figure 1. Arrows highlight the genes upregulated to the highest level in α331 (red arrows) or in col‐0 (blue arrows) when these plants were infected with B05.10 and grown in the presence of ergosterol and squalene at the selected concentration. Three biological replicates were analyzed for each of the conditions used in this experiment. The seven genes included in this study belong to only two groups: (i) salicylic acid (SA)‐related genes (red); (ii) jasmonate/ethylene (JA/ET)‐related genes (green); and were selected from a group of 12 genes related with *A. thaliana* defense and growth (Figure S4).

Real‐time quantitative polymerase chain reaction analysis of the genes related to plant defense and growth revealed that α331 plants showed a markedly different pattern of expression ratios compared with the wild‐type col‐0 plants in seven of the 12 genes analyzed (Figures 6, S4). The B05.10 infection of col‐0 plants grown in the presence of ergosterol/squalene resulted in a higher expression ratio for *PAL*, *JAR*, *AOC*, and *ACS*, compared to plants grown in the absence of these compounds (Figure 6A, B). However, a clearer effect was observed in α331; *PAL*, *ICS*, *EPS1*, and *ACO* showed higher expression ratios in α331 plants grown in the same comparison as above (Figure 6C, D).

The most remarkable differences were observed when comparing the expression ratios of the seven selected genes in α331 plants infected with B05.10 and grown in the presence of ergosterol/squalene, *versus* their expression ratios in col‐0 plants grown in the same conditions. *PAL*, *ICS*, and *EPS1*, all belonging to the SA biosynthetic pathway, as well as *ACO* (ET biosynthesis), were upregulated in the α331 mutant compared to the wild‐type, while *JAR* and *AOC* (JA biosynthesis) and *ACS* (ET biosynthesis) were downregulated in the mutant in the same comparison (Figure 6B, D).

These data indicate that the mutation of the NP_001304843.1 homologous gene in α331, which presumably encodes WRKY40, drastically affected the response of plants to the pathogen B05.10 in the presence of ergosterol and squalene, most likely by modifying their ability to perceive the environmental concentrations of these two compounds.

## DISCUSSION

### Squalene and ergosterol individually affect the transcription level of tomato defense‐related genes, but only ergosterol promotes plant growth

The effect of squalene alone on the expression of tomato defense‐related genes has been already reported (Malmierca et al. 2015c). The main conclusion of that work was that higher concentrations of squalene, such as those produced by the T34‐E20 strain, caused most of the tomato defense genes, except *TomLoxA*, to be downregulated in the leaves compared to their expression in plants grown at lower squalene concentrations, such as those produced by the T34 strain.

To fully explore the effects of ergosterol and squalene, we grew plants in ergosterol without squalene at the concentrations produced by T34 (high concentration) or T34‐E20 (low concentration). The genes *PR‐P2* and *TomLoxA*, which are related to the SA and JA/ET pathways, respectively, were slightly upregulated in plants grown in the higher ergosterol concentration when compared with plants grown without ergosterol. Furthermore, *PR1b1*, *PINI*, and *PINII* were slightly downregulated in plants grown in the higher ergosterol concentration, while *PR‐P2* and *TomLoxA* were not significantly affected, when compared against the condition with the lowest ergosterol concentration (Figure S5).

No remarkable phenotypic changes were observed in plants grown in the two concentrations of squalene relative to the control plants (Malmierca et al. 2015c); however, in the plants grown in the highest (T34) concentration of ergosterol, more secondary roots and thicker main roots were produced than those in plants grown in the lower ergosterol concentration or the control condition lacking ergosterol (Figures S5, S6).

Taken together, these data lead us to conclude that both compounds are important for fungus–plant interactions, including the subsequent regulation of plant growth and the defense responses. Further studies should search for plant genes and proteins allowing plants to perceive and respond to the different levels of ergosterol and squalene present in the fungal membranes.

### Growth on high ergosterol + low squalene concentrations trigger the upregulation of genes related to the JA/ET and to the photosynthetic activity in the tomato roots

The concentrations of these two compounds detected in the *T. harzianum* CECT 2413 (T34) strain were selected based on their positive effect on plant growth (Figures 2, S1). Their lack of effect on the expression of *TomLoxA*, a gene controlling the spread of beneficial fungi in the roots (León‐Morcillo et al. 2012; Harel et al. 2014), suggests that the T34 ergosterol/squalene concentration ratio did not affect the susceptibility of plants to fungal colonization; however, the concentrations of ergosterol and squalene used in this work were significantly different from those deduced for some of the most common fungal pathogens (Malmierca et al. 2015c), suggesting that their concentrations might allow plants to identify a particular fungus as a pathogen (Malmierca et al. 2015c).

The growth of tomato plants treated with ergosterol/squalene, at T34‐induced levels, resulted in an upregulation of tomato *PINI* and *PINII* (ET/JA‐related) genes in the roots (Figure 1), which is a similar result to that previously described in plants infected with B05.10 (Alonso‐Ramírez et al. 2014). Therefore, these compounds would trigger the tomato plant defense response, indicating that they can determine the type of response to be activated, that is the SA or ET/JA pathways.

Furthermore, the expression of *PINI* and *PINII*, encoding proteinase inhibitors, is highly dependent on the level of squalene in the growth medium or in the fungal mycelium, with an increase in the concentration of squalene usually resulting in a downregulation of these genes (Malmierca et al. 2015c). The upregulation of these two genes observed in the current study is consistent with the low concentration of squalene used, which corresponds to the levels in the T34 membranes. These data further emphasize the role of squalene, in addition to ergosterol, in the regulation of plant defense‐related genes, and are consistent with previous reports indicating that the relative concentration of these two compounds in the fungal membrane might allow plants to identify a fungus as a potential pathogenic organism (Cardoza et al. 2015; Malmierca et al. 2015c).

*ACCS*, encoding 1‐aminocyclopropane 1‐carboxylate (ACC) synthase, was downregulated in tomato plants grown in the presence of ergosterol/squalene. ACC is a precursor of ET; therefore, this result is consistent with the reduction in ET observed in other plant‐symbiotic interactions (Vierheilig et al. 1994) and suggests that the reduction in ET levels, presumably as a result of the *ACCS* downregulation, would be involved in the perception of ergosterol/squalene, at the levels produced by T34, as MAMPs belonging to a nonpathogenic fungus. *ACCO* (encoding ACC oxidase) was slightly upregulated in the presence of ergosterol and squalene, but not to a level that would compensate for the downregulation in *ACCS*. The production of ET is therefore most likely slightly reduced in plants inoculated with T34 or grown with the ergosterol/squalene concentration produced from this fungal strain, although it should be present at sufficient levels to act as a signal to induce *PINI* and *PINII* expression, as shown above.

Our analysis of the expression of tomato genes related to plant growth revealed that the ergosterol/squalene concentration used did not significantly affect the expression of *GAI* and *SUCS*, which was consistent with the minor differences in growth observed between plants grown with the ergosterol/squalene concentrations of T34 and without these compounds, at least in the growth conditions initially used (Figure S1A, B). The pattern of *PEPC*, *PEPCK*, *NR*, and *Glb1* expression indicated that the concentrations of ergosterol/squalene selected in these studies trigger the upregulation of photosynthetic activity with no effect on the nitrogen assimilation efficiency.

### The addition of T34 mycelium to the media had similar effects on growth and in the regulation of tomato defense‐related genes than those observed when pure ergosterol + squalene were used at the concentrations produced by this fungal strain

The use of media amended with freeze‐dried T34 and T34‐E20 mycelia enabled us to conclude that their effects on plant growth and on the expression of plant defense‐related genes were similar to those discussed above when using the pure compounds. Furthermore, these results were also confirmed using an *in vivo* experiment in which tomato plants were grown in a commercial soil amended with the same fungal mycelia used in the *in vitro* experiments, which provided further evidence that these compounds act as MAMPs or PAMPS, as is already known to be the case for ergosterol in some phytopathogenic fungi (Nürnberger et al. 2004; Klemptner et al. 2014). Thus, these compounds would be perceived by plant receptors, that is PRRs, and, based on these results, it seems likely that higher concentrations of ergosterol or squalene present in the mycelium of a particular fungus resulted in a higher ability to colonize and stimulate root growth (Malmierca et al. 2015c). Furthermore, *T. harzianum* mutants producing low levels of squalene were previously shown to induce a stronger SA‐related response and have a lower ability to colonize plant roots, indicating that these low squalene‐producing strains are perceived as quasi‐pathogens (Malmierca et al. 2015c).

A comparison of the expression level of plant defense‐related genes when both ergosterol and squalene were present *versus* those experiments where only squalene (Malmierca et al. 2015c) or ergosterol were used resulted in our identification of a complex pattern. Thus, the expression of SA‐related genes in the presence of both compounds follows a pattern similar to that observed when using only ergosterol; however, a drastic downregulation of *PINII* (a JA/ET‐related gene) was observed in the presence of both compounds (see Figure 2), which was clearly different from the pattern observed when ergosterol or squalene were separately analyzed. These data indicate that, in the regulation of SA‐related genes, the effect of ergosterol prevails over that of squalene, while in the regulation of the JA/ET‐related genes, a sort of synergism is established, and neither of these compounds prevails over the other. This pattern emphasizes the importance of this study, in which we analyzed the effect of both compounds together instead of as single amendments.

### Identification of tomato genes encoding for two LRR and one WRKY domain‐containing proteins that are upregulated in the presence of ergosterol/squalene at T34 concentrations, and analysis of *A. thaliana* mutants deleted homologous to these genes

The RNA‐seq analysis of tomato plants grown in the presence of ergosterol/squalene compared to those grown in the control condition led to the identification of two highly DEGs encoding LRR domain‐containing proteins (accession numbers XP_004250531.1 and XP_004238856.2) and another encoding a WRKY domain‐containing protein (NP_001304843.1), all of which could be involved in the plant response mediated by ergosterol and/or squalene. *Arabidopsis thaliana* mutants with deletions in the genes homologous to these three selected tomato genes, α666C, α987C (LRR mutants), and α331 (WRKY mutant), were analyzed, all of which showed phenotypic and transcriptomic differences when compared to the col‐0 wild‐type plants (Figures 4, 5). The α331 mutant exhibited the strongest differences to col‐0; in this line, two of the four SA‐related genes analyzed were drastically upregulated, while all the JA‐ and ET‐related genes analyzed were downregulated, when compared to the expression ratios detected in col‐0 plants. These data indicate that α331 showed a certain degree of desensitization to ergosterol and squalene. Despite the slightly increased growth rate of α331 in the presence of ergosterol and squalene relative to the control condition or to the col‐0 plants, the *PR* genes, *BIK* and *NPR1*, were not significantly affected in col‐0 ecotype by the presence of ergosterol and squalene, indicating that these compounds would not initially be perceived as PAMPs. Furthermore, the upregulation of at least one of these two genes in α666C and α987C, would imply an almost antagonistic role for the PRR and WRKY proteins in the response to ergosterol and squalene in the control of *PR* gene expression.

### *Arabidopsis thaliana* WRKY40‐like protein acts as a transcription factor regulating the ergosterol/squalene effect on genes related to SA and JA/ET pathways in the presence of *B. cinerea*

The *WRKY* gene, which was mutated in α331 strain, was predicted to encode a WRKY40‐like protein. This transcription factor has previously been reported to be involved in the activation of plant defense‐related genes by fungal pathogens (Xu et al. 2006; Shen et al. 2007). WRKY40 is also involved in the responses to abscisic acid and abiotic stress (Chen et al. 2010), but its relationship with the signaling pathway activated by ergosterol and squalene has not been reported. The α331 mutant exhibited a response totally different from that observed in the col‐0 plants when infected with *B. cinerea* in the presence of ergosterol/squalene. The most remarkable difference between both strains was the lack or only slight effect on the expression ratio of JA‐related (*JAR, AOC*) and ET‐related (*ACS*) genes in α331 plants infected with *B. cinerea* and grown in media amended with ergosterol/squalene *versus* plants noninfected with the pathogen, when compared with the values observed for the wild‐type ecotype. In that mutant, genes involved in SA biosynthesis (*PAL*, *ICS*, and *EPS1*) and other related to ET (*ACO*) were upregulated compared to the col‐0 plants grown under the same conditions. The *A. thaliana WRKY40* homolog therefore likely acts as a repressor of the SA‐related pathway and as a partial inducer of the JA/ET‐related genes, which is consistent with data reported for other WRKY TFs, such as WRKY70 (Li et al. 2004).

## CONCLUSION

In summary, this study indicates the following. (i) The relative concentration of ergosterol/squalene in the fungal mycelia affects plant growth and defense responses during fungus–plant interactions. Higher ergosterol concentrations usually resulted in increased plant growth and development, mainly at the root level, and in the upregulation of genes involved in the JA/ET signaling pathways, while the SA pathway genes were not significantly affected in tomato plants. The ergosterol/squalene ratio itself, rather than the concentrations of the individual compounds, was the critical parameter determining these plant responses. (ii) The *A. thaliana* WRKY40 TF is involved in the plant defense response and was activated in response to the perception of fungal ergosterol and squalene. (iii) WRKY40 negatively regulates the expression of SA‐related genes and upregulates some ET/JA‐related genes in the presence of ergosterol and squalene at the T34 concentrations, which was also a similar response to that observed in plants infected with the pathogen *B. cinerea* B05.10.

## MATERIALS AND METHODS

### Strains and culture conditions

*Trichoderma harzianum* CECT 2413 (T34) was used as a reference to compare the levels of ergosterol and squalene production. T34‐E20 (Cardoza et al. 2006), T34‐E1.33 (Cardoza et al. 2014), and T34‐SIL.E7 (Malmierca et al. 2015c) are three derivatives of T34 isolated in previous works that are known to be affected in different steps of the ergosterol biosynthetic pathway. These fungal strains were grown on PPG medium (2% mashed potatoes (Maggi), 2% glucose (Panreac Applichem), and 2% agar (Oxoid Ltd.)) and incubated at 28°C for 4 d to sporulate.

For ergosterol and squalene quantification, strains were grown in potato‐dextrose broth medium (Difco) for 48 h as previously described (Lindo et al. 2019), and the concentrations of these two compounds were determined using procedures widely reported (Cardoza et al. 2007; Ghimire et al. 2009).

To obtain freeze‐dried mycelia from T34 and T34‐E20, 1 × 10^6^ spores/mL of these strains was inoculated in CM broth (0.5% malt extract (Oxoid Ltd.), 0.5% yeast extract (Difco), and 0.5% glucose) and incubated for 24 h at 250 rpm and at 28°C. The mycelia were then harvested by filtration and freeze‐dried. Later, the dry mycelia were ground to powder and used to supplement the MS medium (Sigma Aldrich) used for tomato growth. The MS medium amended with fungal mycelia was heat‐sterilized before use.

*Botrytis cinerea* B05.10, which was isolated from a vineyard (Quidde et al. 1998), was used for the pathogenic bioassays. This fungus was grown on V8 media plates (Oxoid Ltd.) and incubated at 21°C for 8 d to sporulate.

*Solanum lycopersicum* var. Marmande was used for the tomato plant assays.

*Arabidopsis thaliana* ecotype col‐0 was used as the wild‐type control for the experiments performed using the following three mutant plants: SALK_064666C (=α666C), SALK_129987C (=α987C), and SALK_0398331 (=α331). The *A. thaliana* mutants were purchased from the Nottingham Arabidopsis Stock Center (NASC) (http://www.arabidopsis.info).

### Plant assays

#### Tomato assays

##### Growth of tomato plants on media supplemented with pure ergosterol/squalene

Tomato seeds were surface‐sterilized with 70% ethanol and 2.5% sodium hypochlorite and then germinated on MS medium + 0.8% agar on 150‐mm plates and grown for 1 week in a growth chamber at 25°C and 50% relative humidity with a photoperiod of 16 h light/8 h dark. The plants were then transferred (four tomato plants per plate) to other 150‐mm plates containing the following: (i) (control condition) 100 mL MS + 1.5% agar supplemented with 300 µL of Tween:ethanol (1:1) and 200 µL of acetone; (ii) 100 mL MS + 1.5% agar with ergosterol and squalene at the concentrations produced by the T34 strain (Table 1), dissolved in the same volume of solvents used in the control condition; (iii) 100 mL MS + 1.5% agar supplemented with ergosterol and squalene at the concentrations produced by the T34‐E20 strain, a mutant isolated from T34 silenced in the *erg1* gene, encoding the squalene epoxidase (Cardoza et al. 2006); (iv) 100 mL MS + 1.5% agar with ergosterol and squalene at concentrations corresponding to the T34‐E1.33 strain, a T34 mutant strain overexpressing *erg1* (Cardoza et al. 2014); (v) 100 mL MS + 1.5% agar with ergosterol and squalene concentrations corresponding to the T34‐SIL.E7 strain, a T34 mutant strain with the *erg7* gene silenced, a gene encoding an oxidosqualene cyclase (= lanosterol synthase) (Malmierca et al. 2015c); or (vi) 100 mL MS + 1.5% agar amended only with ergosterol at the concentrations produced by T34 or to T34‐E20. The plants were grown for 6 d in the same conditions indicated above. Each condition was tested in triplicate.

Growth of tomato plants on media supplemented with T34 or T34‐E20 mycelia: Tomato plants grown from sterilized seeds were transferred to 150‐mm plates containing 100 mL MS + 1.5% agar and supplemented with 0.4 g of freeze‐dried T34 mycelia (high level of ergosterol; low level of squalene) or T34‐E20 mycelia (low level of ergosterol; high level of squalene). The plants were incubated for 21 d under the conditions described above, and the aerial parts of these plants were collected for RT‐qPCR analysis.

*In vivo* assays: Surface‐sterilized tomato seeds were sown in pots containing 250 mL of commercial loamy field soil (Kekkilä 50/50; Projar S.A.) (Malmierca et al. 2012) amended with 0.01, 0.1, and 0.4 g of T34 or T34‐E20 freeze‐dried mycelia per pot. The pots were incubated in a glasshouse at 21 ± 2°C with a photoperiod of 16 h light/8 h dark and watered as needed. After 4 weeks of growth, the leaves were collected to extract RNA for the RT‐qPCR analysis.

#### *A. thaliana* assays

*Arabidopsis thaliana* seeds were sterilized using 70% ethanol, 0.1% Triton X‐100, and 2.5% sodium hypochlorite and grown on 90‐mm plates containing 25 mL of MS media + 0.8% agar in a growth chamber at 25°C and 50% relative humidity with a photoperiod of 16 h light/8 h dark. Two‐week‐old *A. thaliana* plants were transferred to other 90‐mm plates containing 25 mL of MS media + 1% agar and one of the following: (i) 264 µL of ergosterol (stock 12.5 mg/mL in Tween:ethanol (1:1)) and 71 µL of squalene (stock 0.84 µg squalene/mL in acetone); or (ii) the control condition, in which only the solvents (264 μL Tween:ethanol (1:1) and 71 μL acetone) were added.

After 7 d of incubation, the plants were analyzed to observe the differences in their aerial parts and in their main and secondary root lengths. RNA was extracted from the aerial parts for use in RT‐qPCR analysis. Note that three biological replicates were used for all *A. thaliana* growth experiments.

#### Infection of *A. thaliana* plants with *B. cinerea* B05.10

A 5‐mL aliquot of a suspension of 10^3^ *B. cinerea* B05.10 spores/mL was spread on the surface of plates containing 2‐week‐old *A. thaliana* plants. This suspension was allowed to dry, and the plants were then transferred to another 90‐mm Petri dish containing MS + 1% agar amended with ergosterol and squalene at the concentrations produced by T34 or containing only the solvents (=control condition), as indicated above. Plants were grown for 96 h in this second stage before being harvested for the transcriptomic analysis.

### Nucleic acid extraction and manipulation

RNA was isolated from the roots (tomato) and/or the aerial parts (tomato and *A. thaliana*) following previously described procedures (Malmierca et al. 2015b). The RNA was purified using an RNA Clean & Concentrator kit (Zymo Research), and the complementary DNAs (cDNAs) were synthesized using the iScript cDNA synthesis kit (Bio‐Rad Laboratories), following the manufacturer's instructions.

### RT‐qPCR

The oligonucleotides used for the RT‐qPCR analysis are listed in Table S2. The RT‐qPCR reactions were performed in a final volume of 20 µL using Express SYBR Green RT‐qPCR Super‐mix (Applied Biosystems) following the manufacturer's instructions. The reactions were run in a Step One System (Applied Biosystems), and a comparative and statistical analysis was performed using REST 2009 software (Pfaffl et al. 2002) with the actin‐encoding gene used as housekeeping for the comparative analysis. Three biological replicates with four plants per replicate of each experiment were used for RT‐qPCR quantification.

In the tomato analyses, four different groups of genes were analyzed. (i) Genes involved in the SA signaling pathway, including *ICS* (encoding isochorismate synthase, which is involved in the biosynthesis of isochorismate, a precursor of the SA biosynthetic pathway), *PR1b1*, and *PR‐P2* (Tucci et al. 2011). (ii) Genes belonging to the JA/ET signaling pathway, including *PINI*, *PINII*, *TomLoxA* (Tucci et al. 2011), and *AOC* (encoding the enzyme allene oxide cyclase), involved in ethylene biosynthesis. *ACCS* and *ACCO* were also examined, which encode two enzymes involved in the conversion of ACC (1‐aminociclopropane‐1‐carboxilic acid) to ethylene. ACCS is the ACC synthase, which synthesizes ACC as a derived compound of the methionine cycle, and ACCO (ACC oxidase) removes the carboxylic group from ACC to form ethylene. (iii) Genes involved in growth and development, including *SUCS* (encoding sucrose synthase) and *GAI*, the product of which is a DELLA protein growth repressor. (iv) Genes related to nitrogen metabolism, such as *PEPC* (encoding phosphoenolpyruvate carboxylase), *PEPCK* (encoding phosphoenolpyruvate kinase), *NR* (encoding nitrate reductase), and *Glb‐1* (involved in the detoxification of nitric oxide to nitrate using O_2_) (Buchanan et al. 2015).

In *A. thaliana*, five groups of genes were analyzed using RT‐qPCR. (i) Genes related to the SA signaling pathway, including *PAL* (encoding phenylalanine ammonia‐lyase, which is involved in the biosynthesis of SA from phenylalanine), *PBS3* (an auxin‐responsive GH3 family protein, acting as a positive regulator of SA biosynthesis), *ICS* (isochorismate synthase, the first gene involved in the biosynthesis of SA from shikimic acid), and *EPS1* (encoding an acyltransferase belonging to the BAHD family), all of which were involved in different steps of the SA biosynthetic pathway or in the regulation of its biosynthesis. (ii) Genes involved in JA biosynthesis, including *JAR* (jasmonate‐amido synthetase, a member of the auxin GH3 family of proteins catalyzing the formation of the bioactive jasmonyl‐isoleucine conjugate, downstream of JA biosynthesis) and *AOC* (allene oxide cyclase). (iii) Genes belonging to the ET pathway, including *ACS* and *ACO*. (iv) *PR* genes, including *NPR1* (which encodes a systemic acquired response regulatory protein) and *BIK* (encoding *BOTRYTIS*‐INDUCED KINASE1, a negative regulator of SA accumulation involved in the plant defense against *Botrytis* sp., *Alternaria brassicicola*, and bacterial pathogens). (v) Genes involved in plant growth regulation, including *GAI* (encoding a DELLA protein) and *IAA* (encoding an indoleacetic acid‐induced protein acting as a transcriptional repressor of the auxin response and an inducer of secondary root formation) (Buchanan et al. 2015).

### Tomato RNA‐seq

One‐week‐old tomato plants were grown in selected conditions: (i) 100 mL MS + 1.5% agar with 300 µL of Tween:ethanol (1:1) and 200 µL of acetone (= control condition) or (ii) 100 mL MS + 1.5% agar amended with ergosterol and squalene at the concentrations calculated for T34 (see Table 1), dissolved in the same volume of solvents as in the control condition. The messenger RNAs were extracted and purified from these samples, and the cDNAs were synthesized as indicated above. Two biological replicates were pooled from each sample. The two resulting cDNA libraries were sequenced using the Ion Torrent Ion‐Proton Sequencer platform (Thermo Fisher Scientific). The reads were mapped against the *S. lycopersicum* genome using Tophat2 v2.1.0 (Trapnell et al. 2009), and high‐quality reads were selected and assembled using cufflinks v2.2.1 (Trapnell et al. 2010). The gene quantitation was performed using htsq_count 0.6.1p1 (Anders et al. 2015). A total of 120,166,032, and 115,359,476 reads were obtained from the control and ergosterol/squalene samples, respectively, among which 94.96% and 96.2% were successfully mapped against the *S. lycopersicum* annotated genome. A statistical analysis was performed in R (R Core Team 2018) to check that the two biological replicates could be used as dependent samples, and the differential expression between both samples was performed with Phyton and R software, using the DESeq. 2 algorithm (Anders and Huber 2010). Finally, a hypergeometric test was performed using the Uniprot, Basic Local Alignment Search Tool‐x, and EuKaryotic Orthologous Groups databases, and a functional category was assigned to each sequence with a statistical significance of *P* = 0.05.

### Statistical analysis

IBM SPSS Statistics 24 software was used for the statistical analysis. A comparison of three groups was performed using an analysis of variance with Tukey test, while comparisons of two groups were performed using Student's *t*‐test.

## AUTHOR CONTRIBUTIONS

S.G., L.L., and R.C. conceived the study, designed the experiments, coordinated the experiments with the other co‐authors, analyzed the RT‐qPCR data, and wrote the manuscript. P.C. and A.L. conducted the tomato plant experiments, wrote the corresponding sections of the manuscript, and contributed to editing the entire manuscript. L.L. and R.C. also performed RT‐qPCR analyses and quantified the ergosterol and squalene concentrations. All authors read and approved the paper.

## Supporting information

Additional Supporting Information may be found online in the supporting information tab for this article: http://onlinelibrary.wiley.com/doi/10.1002/jipb.12862/suppinfo

**Figure S1.** Tomato plants growing in Murashige–Skoog media with different concentrations of ergosterol/squalene
(**A**) Control plants grown without ergosterol/squalene. (**B**–**E)** Plants with ergosterol/squalene concentrations corresponding to those produced by strains T34 (**B**), T34‐E20 (**C**), T34‐E1.33 (**D**) or to the T34‐SIL.E7 (**E**).
**Figure S2**. Photographs of roots detached from tomato plants grown for 4 weeks in commercial loamy field soil amended with different amounts of T34 or T34‐E20 freeze‐dried mycelia, as indicated in the caption to Figure 3
**Figure S3**. (**A**, **C**) Plants infected with B05.10 and grown in Murashige–Skoog medium without ergosterol/squalene. (**B**, **D**) Plants infected with B05.10 and grown in media amended with ergosterol/squalene. (**A**, **B**) *Arabidopsis thaliana* col‐0 plants. (**C**, **D**) α331 mutant.
**Figure S4**. Assessment of the response of the α331 mutated ecotype against *Botrytis cinerea* in the presence of ergosterol/squalene, compared to the *Arabidopsis thaliana* col‐0 (wild‐type)
Quantitative polymerase chain reaction (qPCR) analysis of the expression of 12 *A. thaliana* genes in plants infected with *B. cinerea* B05.10 and grown with or without ergosterol and squalene
(**A**, **C**) Expression ratios of the analyzed genes in plants infected with B05.10 and grown in Murashige–Skoog medium without ergosterol/squalene *versus* the levels of expression in plants noninfected with B05.10 and grown in the same conditions. (**B**, **D**) Expression ratios of the analyzed genes in plants infected with B05.10 and grown in media amended with ergosterol/squalene *versus* plants grown in the same conditions but noninfected with B05.10. (**A**, **B**) *A. thaliana* col‐0 plants. (**C**, **D**) α331 plants. Analysis of qPCR data was performed using the REST© software (Pfaffl et al. 2002). Statistically significant values (p(H1) < 0.05) are indicated with an asterisk in the tables below the graphs, and squared with a dotted rectangle in the graphical representation. Four groups of genes were analyzed: (i) salicylic acid (SA)‐related genes (red); (ii) jasmonate/ethylene (JA/ET)‐related genes (green); (iii) pathogenesis‐related genes (pink); and development‐related genes (orange). Pfaffl MW, Horgan GW, Dempfle L (2002) Relative expression software tool (REST) for group‐wise comparison and statistical analysis of relative expression results in real‐time PCR. **Nucleic Acids Res** 30:e36.
**Figure S5**. Effect of ergosterol on tomato growth and in expression of plant defense‐related genes
(**A**) Control plants grown in the presence of ethanol:Tween 80 (1:1) (TET) and tomato plants grown in Murashige–Skoog medium supplemented with 0.022 or 0.033 mg of ergosterol per milliliter (E1 and E2, respectively). (**B**) Quantitative polymerase chain reaction (qPCR) analysis of the relative level of expression of five tomato defense‐related genes from these plants. Boxes with solid lines indicate salicylic acid (SA)‐related genes with statistically significant differences; boxes with dotted lines indicate jasmonate/ethylene (JA/ET)‐related genes with statistically significant differences. Values of expression ratios statistically significant are indicated with an asterisk in the tables at the bottom of the figure. Analysis of qPCR data was performed as indicated in the legend to Figure S4. Two groups of genes were analyzed: (i) SA‐related genes (red); (ii) JA/ET‐related genes (green).
**Figure S6**. Enlarged images of roots from tomato plants grown with the concentrations of ergosterol described in Figure S5
Note the increase in number of secondary roots and in the thickness of the main root when the higher concentration of ergosterol was used (T34 concentration = E2) (right panels)
**Table S1**. Concentration of ergosterol/squalene in Murashige–Skoog medium amended with 0.4 g of freeze‐dried mycelia of T34 or T34‐E20*
**Table S2**. Oligonucleotides used in this work
