# Supplementary material for: Identification of plant genes putatively involved in the perception of fungal ergosterol‐squalene
Source: J Integr Plant Biol. 2019 Dec 30;62(7):927–47. doi: 10.1111/jipb.12862 (PMC7383801; doi:10.1111/jipb.12862)
Supplement: Supplementary file 1 — Figure S1. Tomato plants growing in Murashige–Skoog media with different concentrations of ergosterol/squalene (A) Control plants grown without ergosterol/squalene. (B–E) Plants with ergosterol/squalene concentrations corresponding to those produced by strains T34 (B), T34‐E20 (C), T34‐E1.33 (D) or to the T34‐SIL.E7 (E). Figure S2. Photographs of roots detached from tomato plants grown for 4 weeks in commercial loamy field soil amended with different amounts of T34 or T34‐E20 freeze‐dried mycelia, as indicated in the caption to Figure 3 Figure S3. (A, C) Plants infected with B05.10 and grown in Murashige–Skoog medium without ergosterol/squalene. (B, D) Plants infected with B05.10 and grown in media amended with ergosterol/squalene. (A, B) Arabidopsis thaliana col‐0 plants. (C, D) α331 mutant. Figure S4. Assessment of the response of the α331 mutated ecotype against Botrytis cinerea in the presence of ergosterol/squalene, compared to the Arabidopsis thaliana col‐0 (wild‐type) Quantitative polymerase chain reaction (qPCR) analysis of the expression of 12 A. thaliana genes in plants infected with B. cinerea B05.10 and grown with or without ergosterol and squalene (A, C) Expression ratios of the analyzed genes in plants infected with B05.10 and grown in Murashige–Skoog medium without ergosterol/squalene versus the levels of expression in plants noninfected with B05.10 and grown in the same conditions. (B, D) Expression ratios of the analyzed genes in plants infected with B05.10 and grown in media amended with ergosterol/squalene versus plants grown in the same conditions but noninfected with B05.10. (A, B) A. thaliana col‐0 plants. (C, D) α331 plants. Analysis of qPCR data was performed using the REST© software (Pfaffl et al. 2002). Statistically significant values (p(H1) < 0.05) are indicated with an asterisk in the tables below the graphs, and squared with a dotted rectangle in the graphical representation. Four groups of genes were analyzed: (i) salicylic acid (SA)‐r [file JIPB-62-927-s001.pdf]

# Identification of plant genes putatively involved in the perception of fungal ergosterol-squalene

**Laura Lindo[1,2], Rosa E. Cardoza[1,2], Alicia Lorenzana[2], Pedro A. Casquero[2], Santiago Gutiérrez[1,2]***

[1]Area of Microbiology, University of León, Campus of Ponferrada, Ponferrada, Spain
[2] University Group of Research in Engineering and Sustainable Agriculture, University of León, León, Spain.

## *Supplementary Material*

**Table S1**. Concentration of ergosterol/squalene in MS medium amended with 0.4 g of freeze dried mycelia of T34 or T34-E20*

| | Squalene (mg/mL)** | Ergosterol (mg/mL)** |
|---|---|---|
| T34 | 0.006 | 0.089 |
| T34-E20 | 0.013 | 0.054 |

*.- quantitation of ergosterol/squalene were carried out after the media were heat sterilized.

**.- Concentrations are given in mg of ergosterol or squalene per mL of MS medium

**Table S2**. Oligonucleotides used in this work

| Name | Sequence 5'-3' | Name | Sequence 5'-3' |
|---|---|---|---|
| ***S. lycopersicum* genes** | | ***A. thaliana* genes** | |
| *PR-1b1F* | gcactaaacctaaagaaaaatggg | *ACSF* | cgaagattgtaaccgcga |
| *PR-1b1R* | aagttggcatcccaagacata | *ACSR* | tgaaccaccctgtcattg |
| *PR-P2F* | ggaacaggaacacaagaaacagtga | *AOCF* | tacttcggagactatggc |
| *PR-P2R* | cccaatccattagtgtccaatcg | *AOCR* | tccgtaggcaccttcaaa |
| *ICSF* | tgcctcatggacataccaga | *GAIF* | accggcaccggataattt |
| *ICSR* | ggcacatgtgtattggcaag | *GAIR* | gccacaaatcctctgtac |
| *ACCSF* | tgagttggtgaaccatggaa | *ICSF* | ccgtccagatcttcttca |
| *ACCSR* | gcttgaacagcctcaagtcc | *ICSR* | gtcgagatcgcggaaaaa |
| *ACCOF* | gccctgcttatcttcgattg | *ACOF* | aatacccagaatgcccac |
| *ACCOR* | ttcgtgcttgatcagaatgc | *ACOR* | aagaactcaagaccaggc |
| *GAIF* | acctccggtgaacaatcaag | *PALF* | aaggtctcgcgctagtcaat |
| *GAIR* | gaacgcatttgaacccagat | *PALR* | gccgacaaaatctcagccaa |
| *SUCSF* | atgaaccgagtgaggaatgg | *PBS3F* | cgaaaccgttgtcactggtt |
| *SUCSR* | gctggaccaccgtgattagt | *PBS3R* | tcaggtttgcacagaggatc |
| *PINIF* | tgaaactctcatggcacgaaaag | *EPS1F* | cgagaggaagagactcattg |
| *PINIR* | ggccacatttgttttccttcg | *EPS1R* | tcccggtttggcttacattc |
| *PINIIF* | ggccaaatgcttgcaccttt | *JARF* | tattactgtcccatcggtcc |
| *PINIIR* | cgtggtacatccggtgggata | *JARR* | cgtaccagttgctcaaactc |
| *TomLoxAF* | tgaaccatggtgggctgaaa | *NPR1F* | ataaggcacttgactcgg |
| *TomLoxAR* | ctgcccgaaattgactgctg | *NPR1R* | cagcgaaatgaagagcac |
| *PEPCF* | cccgacctaccaaaaactga | *IAAF* | agaagcttctcttcccgttg |
| *PEPCR* | tggcattcacgctgtagaag | *IAAR* | tcccaagtgtcagcaaatcc |
| *PEPCKF* | tgccagagcctatcactcct | *BIKIF* | tggatcataacagaccgg |
| *PEPCKR* | aggttacatgggaattgacc | *BIKIR* | gtgtgtctagccgattgt |
| *NRF* | tctcccaataggtgcattcc | | |
| *NRR* | tacctgttccacccgctatc | | |
| *Glb-1F* | tggtgaatggggtctcaaat | | |
| *Glb-1R* | ctgccgcttcacaagtcata | | |

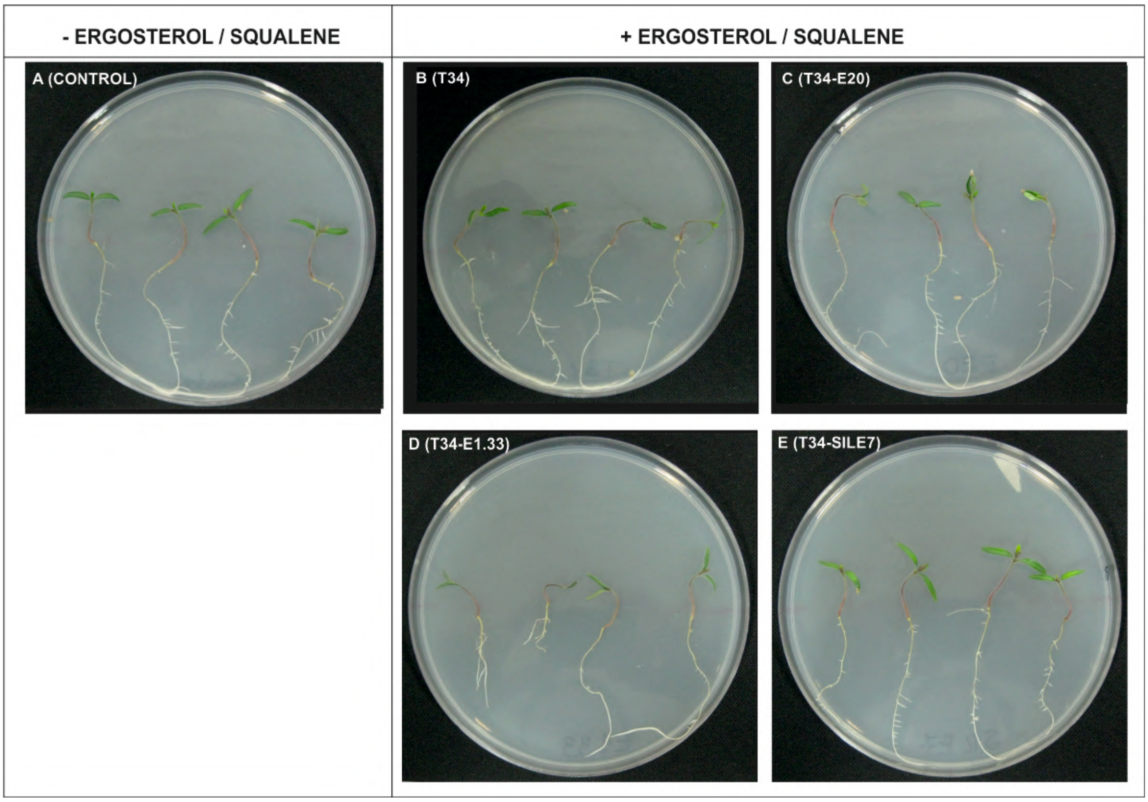


**Figure S1**. Tomato plants growing in MS media with different concentrations of ergosterol / squalene: A.- Control plants grown without ergosterol / squalene; B-E.- plants with ergosterol / squalene concentrations corresponding to those produced by strains T34 (B), T34-E20 (C), T34-E1.33 (D) or to the T34-SIL.E7 (E).

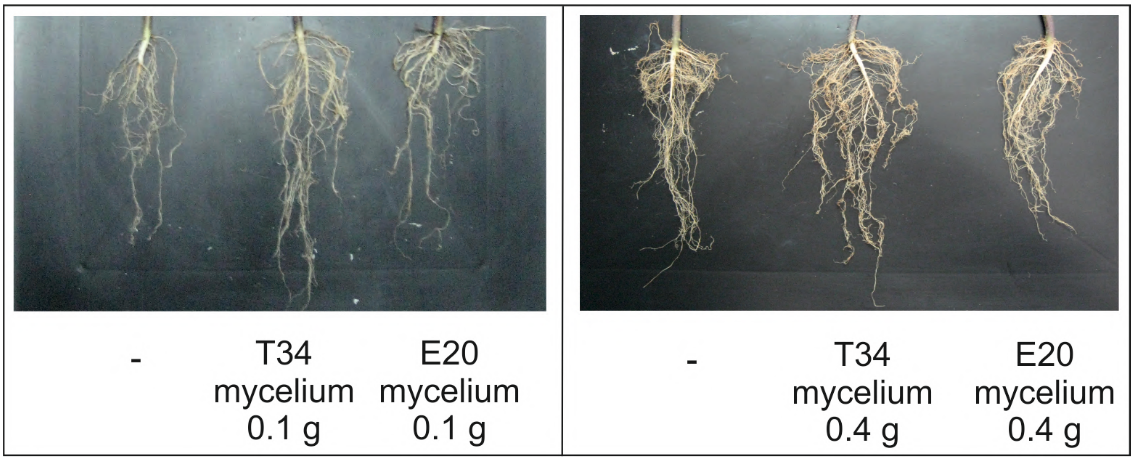


**Figure S2**. Photographs of roots detached from tomato plants grown for four weeks in commercial loamy field soil amended with different amounts of T34 or T34-E20 freeze dried mycelia, as indicated in the caption to Fig. 3.

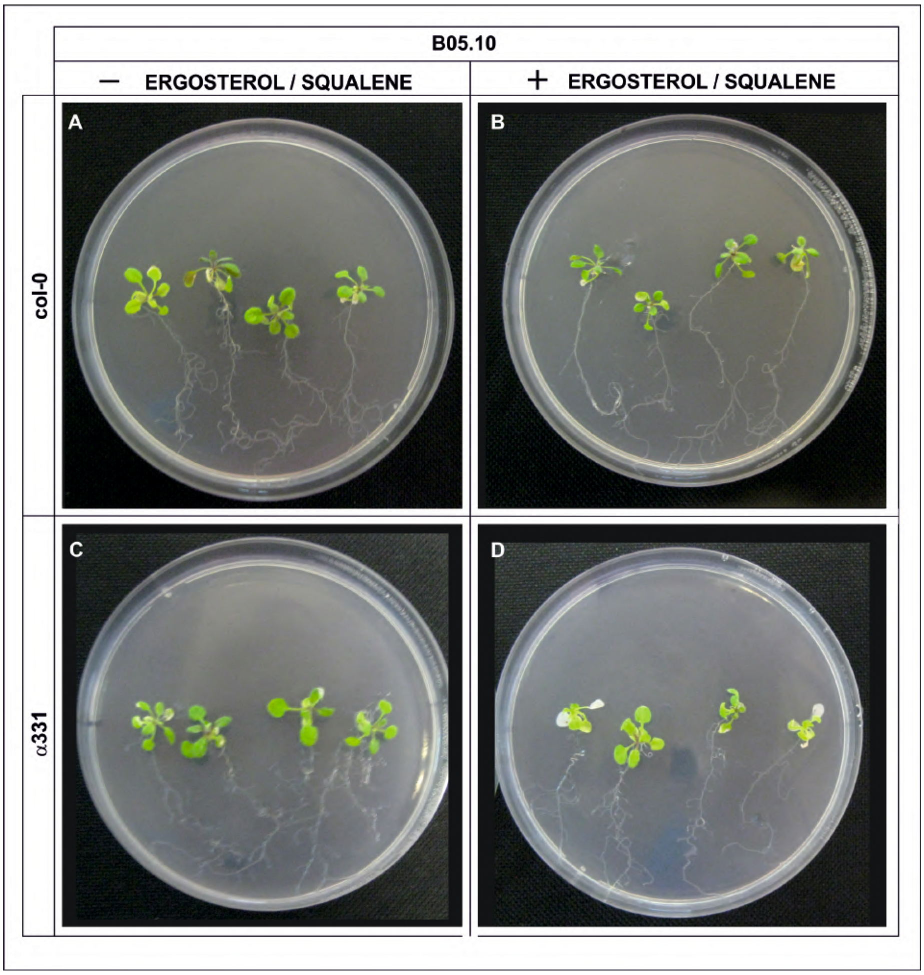


**Figure S3**. A, C.- plants infected with B05.10 and grown in MS medium without ergosterol / squalene. B, D.- plants infected with B05.10 and grown in media amended with ergosterol / squalene. A, B.- *A. thaliana* col-0 plants. C, D.- α331 mutant.

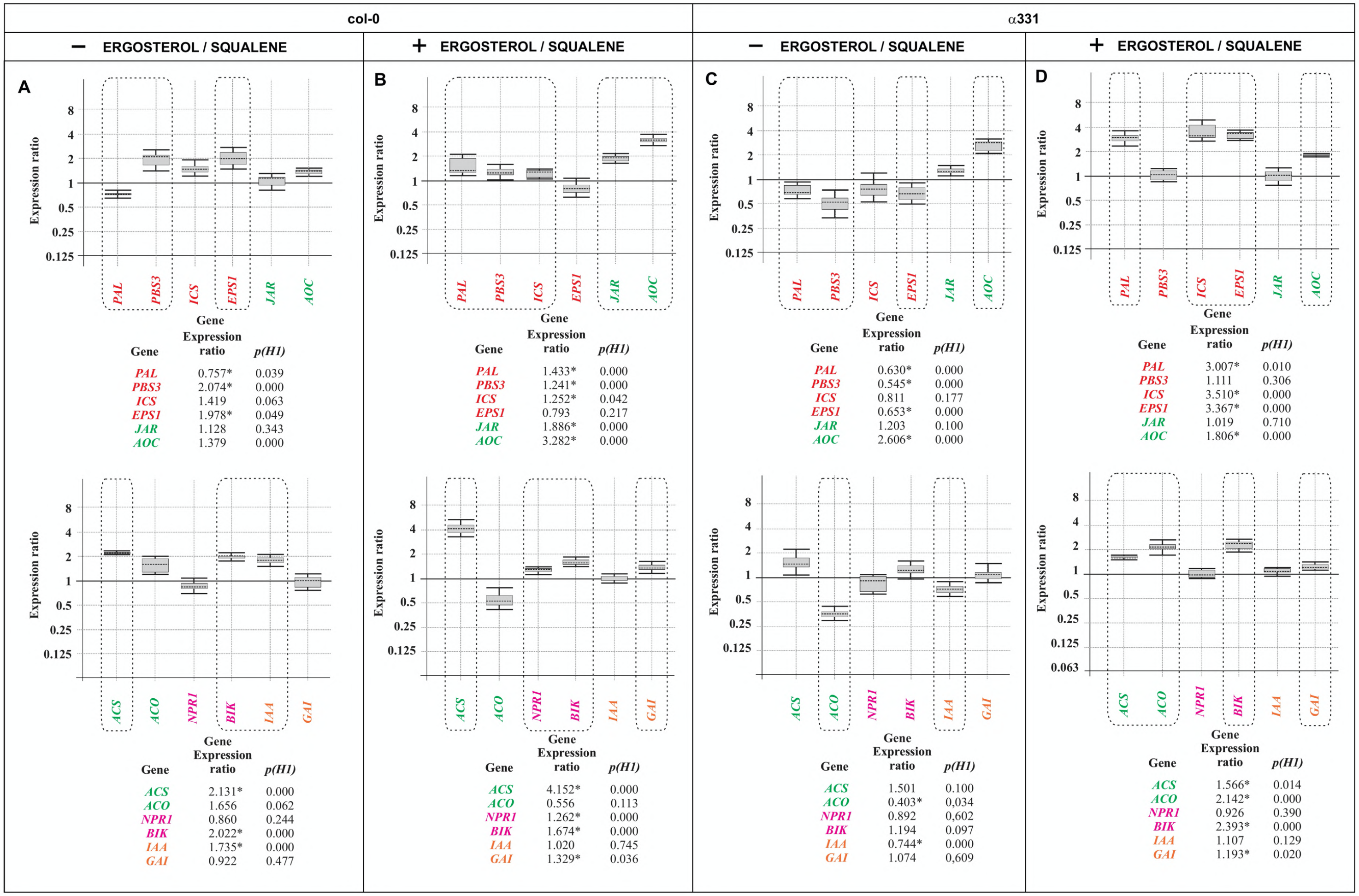


**A (col-0, − ergosterol / squalene)**

| Gene | Expression ratio | p(H1) |
|---|---|---|
| *PAL* | 0.757* | 0.039 |
| *PBS3* | 2.074* | 0.000 |
| *ICS* | 1.419 | 0.063 |
| *EPS1* | 1.978* | 0.049 |
| *JAR* | 1.128 | 0.343 |
| *AOC* | 1.379 | 0.000 |

| Gene | Expression ratio | p(H1) |
|---|---|---|
| *ACS* | 2.131* | 0.000 |
| *ACO* | 1.656 | 0.062 |
| *NPR1* | 0.860 | 0.244 |
| *BIK* | 2.022* | 0.000 |
| *IAA* | 1.735* | 0.000 |
| *GAI* | 0.922 | 0.477 |

**B (col-0, + ergosterol / squalene)**

| Gene | Expression ratio | p(H1) |
|---|---|---|
| *PAL* | 1.433* | 0.000 |
| *PBS3* | 1.241* | 0.000 |
| *ICS* | 1.252* | 0.042 |
| *EPS1* | 0.793 | 0.217 |
| *JAR* | 1.886* | 0.000 |
| *AOC* | 3.282* | 0.000 |

| Gene | Expression ratio | p(H1) |
|---|---|---|
| *ACS* | 4.152* | 0.000 |
| *ACO* | 0.556 | 0.113 |
| *NPR1* | 1.262* | 0.000 |
| *BIK* | 1.674* | 0.000 |
| *IAA* | 1.020 | 0.745 |
| *GAI* | 1.329* | 0.036 |

**C (α331, − ergosterol / squalene)**

| Gene | Expression ratio | p(H1) |
|---|---|---|
| *PAL* | 0.630* | 0.000 |
| *PBS3* | 0.545* | 0.000 |
| *ICS* | 0.811 | 0.177 |
| *EPS1* | 0.653* | 0.000 |
| *JAR* | 1.203 | 0.100 |
| *AOC* | 2.606* | 0.000 |

| Gene | Expression ratio | p(H1) |
|---|---|---|
| *ACS* | 1.501 | 0.100 |
| *ACO* | 0.403* | 0,034 |
| *NPR1* | 0.892 | 0,602 |
| *BIK* | 1.194 | 0.097 |
| *IAA* | 0.744* | 0.000 |
| *GAI* | 1.074 | 0,609 |

**D (α331, + ergosterol / squalene)**

| Gene | Expression ratio | p(H1) |
|---|---|---|
| *PAL* | 3.007* | 0.010 |
| *PBS3* | 1.111 | 0.306 |
| *ICS* | 3.510* | 0.000 |
| *EPS1* | 3.367* | 0.000 |
| *JAR* | 1.019 | 0.710 |
| *AOC* | 1.806* | 0.000 |

| Gene | Expression ratio | p(H1) |
|---|---|---|
| *ACS* | 1.566* | 0.014 |
| *ACO* | 2.142* | 0.000 |
| *NPR1* | 0.926 | 0.390 |
| *BIK* | 2.393* | 0.000 |
| *IAA* | 1.107 | 0.129 |
| *GAI* | 1.193* | 0.020 |

**Figure S4**. Assessment of the response of the α331 mutated ecotype against *B. cinerea* in the presence of ergosterol / squalene, compared to the *A. thaliana* col-0 (wild type). qPCR analysis of the expression of twelve *A. thaliana* genes in plants infected with *B. cinerea* B05.10 and grown with or without ergosterol and squalene. (A, C) Expression ratios of the analyzed genes in plants infected with B05.10 and grown in MS medium without ergosterol / squalene *versus* the levels of expression in plants non infected with B05.10 and grown in the same conditions. (B, D) Expression ratios of the analyzed genes in plants infected with B05.10 and grown in media amended with ergosterol / squalene *versus* plants grown in the same conditions but non-infected with B05.10. (A, B) *A. thaliana* col-0 plants. (C, D) α331 plants. Analysis of qPCR data was performed using the REST© software (Pfaffl et al. 2002). Statistically significant values [p(H1)< 0.05] are indicated with an asterisk in the Tables below the graphs, and squared with a dotted rectangle

in the graphical representation. Four groups of genes were analyzed: (i) SA-related genes (red); (ii) JA/ET-related genes (green); (iii) Pathogenesis related genes (pink); and development related genes (orange).

Pfaffl MW, Horgan GW, Dempfle L (2002) Relative expression software tool (REST) for group-wise comparison and statistical analysis of relative expression results in real-time PCR. **Nucleic Acids Res** 30:e36

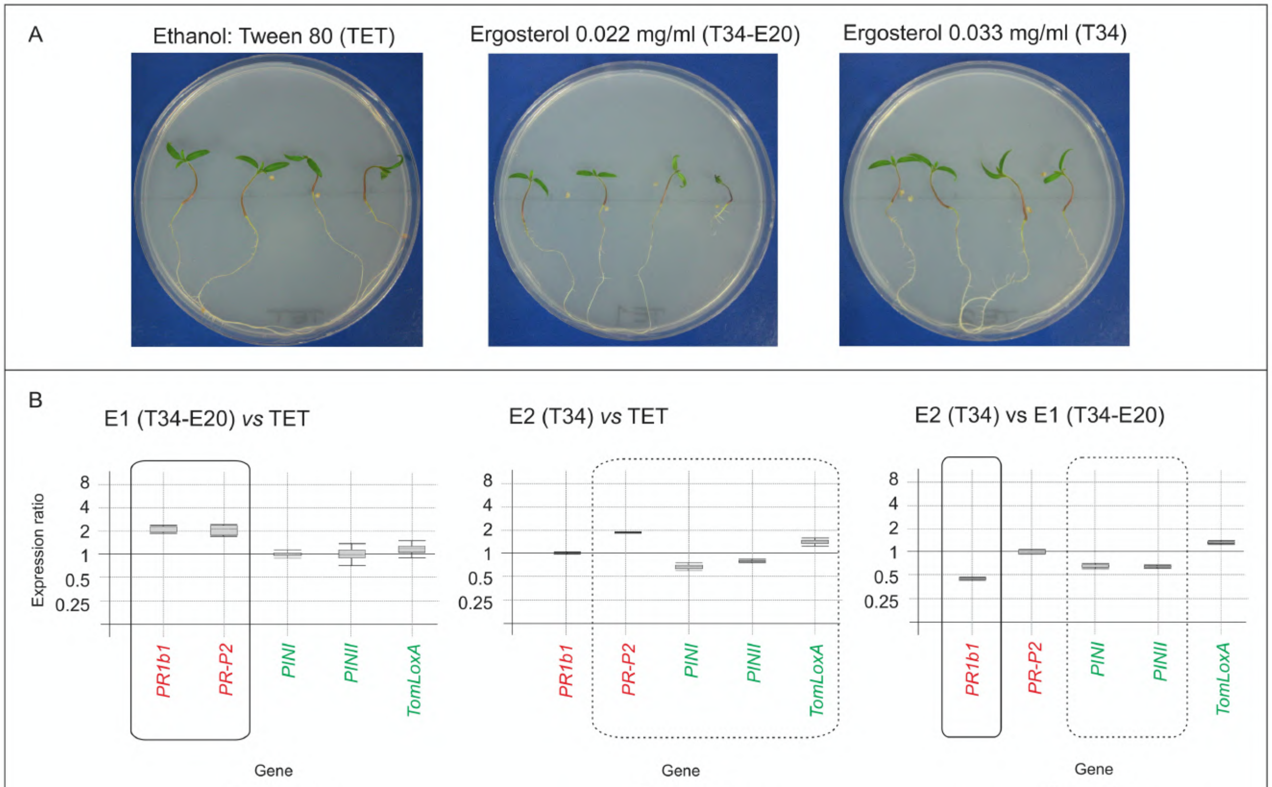


| Gene | Expression ratio | p(H1) |
|---|---|---|
| *PR1b1* | 2.167* | 0.000 |
| *PR-P2* | 2.003* | 0.000 |
| *PINI* | 0.980 | 0.869 |
| *PINII* | 0.975 | 0.808 |
| *TomLoxA* | 1.154 | 0.204 |

| | Expression ratio | p(H1) |
|---|---|---|
| *PR1b1* | 1.006 | 0.515 |
| *PR-P2* | 1.872* | 0.000 |
| *PINI* | 0.669* | 0.000 |
| *PINII* | 0.793* | 0.000 |
| *TomLoxA* | 1.415* | 0.000 |

| Gene | Expression ratio | p(H1) |
|---|---|---|
| *PR1b1* | 0.445* | 0.000 |
| *PR-P2* | 1.001 | 0.834 |
| *PINI* | 0.653* | 0.000 |
| *PINII* | 0.641* | 0.000 |
| *TomLoxA* | 1.329 | 0.173 |

**Figure S5**. Effect of ergosterol on tomato growth and in expression of plant defense-related genes. (A) Control plants grown in the presence of ethanol:tween 80 (1:1) (TET) and tomato plants grown in Murashige and Skoog medium supplemented with 0.022 or 0.033 mg of ergosterol per milliliter (E1 and E2, respectively). (B) Quantitative polymerase chain reaction analysis of the relative level of expression of five tomato defense-related genes from these plants. Boxes with solid lines indicate salicylic acid-related genes with statistically significant differences; boxes with dotted lines indicate jasmonate/ethylene-related genes with statistically significant differences. Values of expression ratios statistically significant are indicated with an asterisk in the tables at the bottom of the figure. Analysis of qPCR data was performed as indicated in the legend to Figure S4. Two groups of genes were analyzed: (i) SA-related genes (red); (ii) JA/ET-related genes (green).

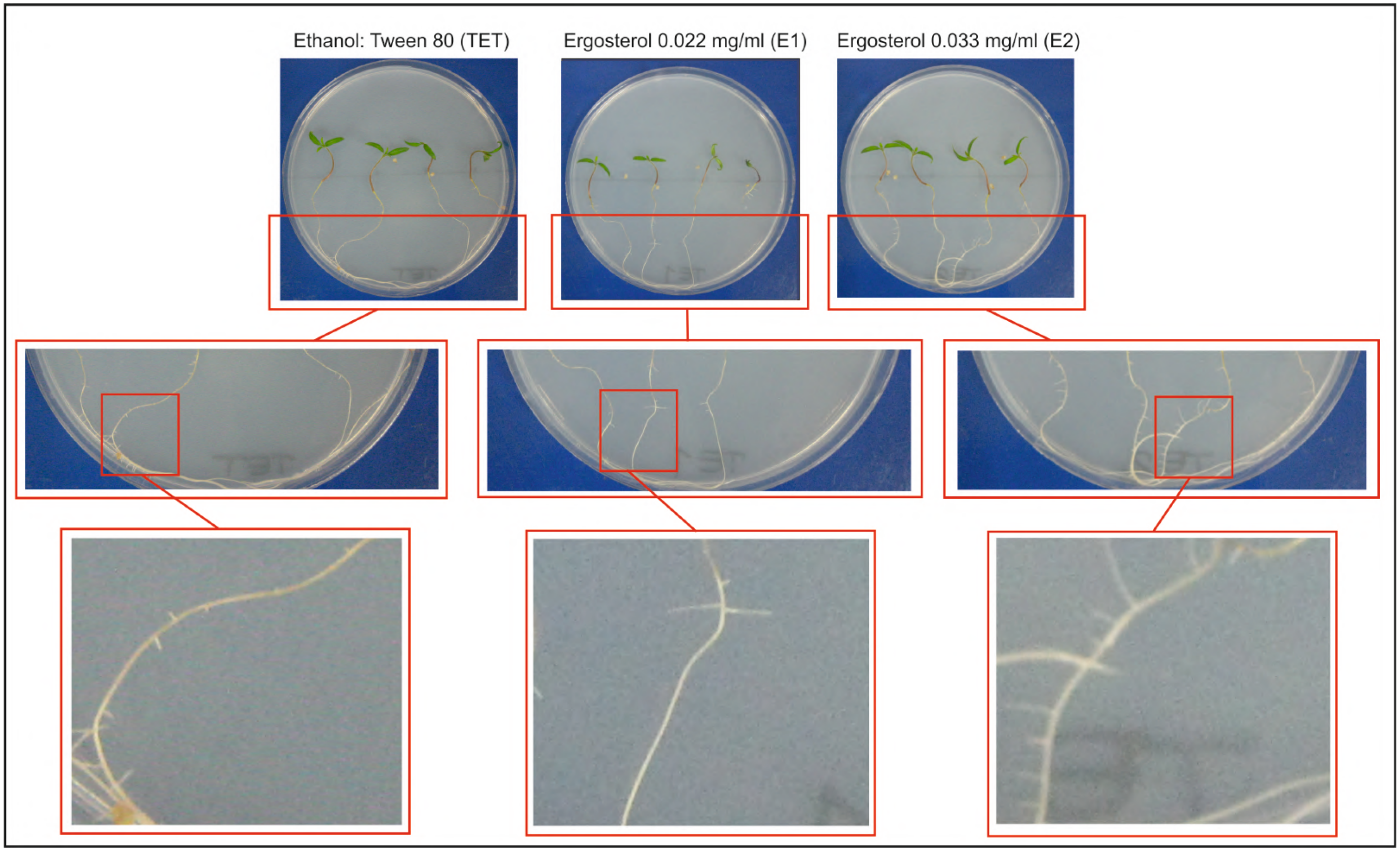


**Figure S6**. Enlarged images of roots from tomato plants grown with the concentrations of ergosterol described in Fig. S5. Note the increase in number of secondary roots and in the thickness of the main root when the higher concentration of ergosterol was used (T34 concentration= E2) (right panels).
